# Analysis of phylogenomic datasets reveals conflict, concordance, and gene duplications with examples from animals and plants

**DOI:** 10.1186/s12862-015-0423-0

**Published:** 2015-08-05

**Authors:** Stephen A Smith, Michael J Moore, Joseph W Brown, Ya Yang

**Affiliations:** Department of Ecology and Evolutionary Biology, University of Michigan, S State St, Ann Arbor, 48109 MI USA; Department of Biology, Oberlin College, W Lorain St, Oberlin, 44074 OH USA

**Keywords:** Phylogenomics, Incomplete lineage sorting, Transcriptome, Gene tree conflict, Gene duplication

## Abstract

**Background:**

The use of transcriptomic and genomic datasets for phylogenetic reconstruction has become increasingly common as researchers attempt to resolve recalcitrant nodes with increasing amounts of data. The large size and complexity of these datasets introduce significant phylogenetic noise and conflict into subsequent analyses. The sources of conflict may include hybridization, incomplete lineage sorting, or horizontal gene transfer, and may vary across the phylogeny. For phylogenetic analysis, this noise and conflict has been accommodated in one of several ways: by binning gene regions into subsets to isolate consistent phylogenetic signal; by using gene-tree methods for reconstruction, where conflict is presumed to be explained by incomplete lineage sorting (ILS); or through concatenation, where noise is presumed to be the dominant source of conflict. The results provided herein emphasize that analysis of individual homologous gene regions can greatly improve our understanding of the underlying conflict within these datasets.

**Results:**

Here we examined two published transcriptomic datasets, the angiosperm group Caryophyllales and the aculeate Hymenoptera, for the presence of conflict, concordance, and gene duplications in individual homologs across the phylogeny. We found significant conflict throughout the phylogeny in both datasets and in particular along the backbone. While some nodes in each phylogeny showed patterns of conflict similar to what might be expected with ILS alone, the backbone nodes also exhibited low levels of phylogenetic signal. In addition, certain nodes, especially in the Caryophyllales, had highly elevated levels of strongly supported conflict that cannot be explained by ILS alone.

**Conclusion:**

This study demonstrates that phylogenetic signal is highly variable in phylogenomic data sampled across related species and poses challenges when conducting species tree analyses on large genomic and transcriptomic datasets. Further insight into the conflict and processes underlying these complex datasets is necessary to improve and develop adequate models for sequence analysis and downstream applications. To aid this effort, we developed the open source software phyparts (https://bitbucket.org/blackrim/phyparts), which calculates unique, conflicting, and concordant bipartitions, maps gene duplications, and outputs summary statistics such as internode certainy (ICA) scores and node-specific counts of gene duplications.

**Electronic supplementary material:**

The online version of this article (doi:10.1186/s12862-015-0423-0) contains supplementary material, which is available to authorized users.

## Background

Genomic and transcriptomic datasets have been instrumental in discerning phylogenetic relationships in major clades that have traditionally proven recalcitrant to phylogenetic resolution when using limited numbers of genes (e.g., [1–10]). The primary goal for many of these studies has been the reconstruction of a species trees where the accumulation of signal from hundreds or thousands of genes provides enough information to overcome phylogenetic noise and uncertainty in resolving relationships. Despite these successes, and with few exceptions [3, 9, 11, 12], there has been little exploration of the distribution of topological conflict and concordance among individual gene tree histories. Instead, the conflict among trees constructed using alternative methods (e.g., concatenation and coalescence) and subsets of a larger dataset are typically explored (e.g.,[8, 10]). As transcriptomic and genomic datasets become increasingly common, it is imperative that we begin to explore conflicting signals among gene trees not only to better elucidate species trees, but also because such conflict itself may be a window into the molecular evolution of the genome. Furthermore, by better understanding the conflict within these analyses, we can potentially better model the processes that generate discordance.

The potential sources of conflict among gene trees may include, but are not limited to, hidden paralogy, hybridization, incomplete lineage sorting (ILS) due to rapid radiation and/or recent divergence, lack of signal due to saturation, recombination, and horizontal gene transfer [13]. For traditional datasets consisting of relatively few loci, a number of methods have been developed to accommodate these processes, although individual methods typically target a single source of conflict. In particular, there are sophisticated methods that have been developed based on coalescent theory to address the problem of incomplete lineage sorting (e.g., [14–19]). These methods are commonly applied to phylogenomic datasets with the goal of resolving a species tree and with the presumption that incomplete lineage sorting underlies the difficulty in resolving recalcitrant nodes (e.g., [8–10]). Further exploration of the conflicting nodes is not often pursued. Other methods that explicitly address topological concordance include concordance analysis as implemented in BUCKy [14, 20, 21]. These and other analyses are limited in a number of ways (e.g., do not scale well with dataset size, are restricted to analyzing groups of orthologous sequences, and do not straightforwardly deal with partially overlapping taxon sets across loci).

In the past two years, several new methods have been developed to address problems in gene tree/species tree reconciliation specifically in phylogenomic datasets. These include a binning procedure meant to address the combination of weak signal from individual genes together with genuine conflicting histories across genes due to ILS [19, 22], a filtering procedure meant to exclude genes with low signal [23], as well as a joint gene tree/species tree estimation procedure [24]. Additionally, there have been efforts to better characterize the uncertainty and conflict at internal edges within these datasets. [25] describe a new measure that calculates the distribution of conflict among alternative topologies, and [26] explore a simple gene jackknife to examine sensitivity of gene inclusion. While these first steps are promising, these methods take into account only a subset of the potential sources of conflicts, and efforts to accommodate multiple sources of conflict, such as accommodating ILS with gene duplications [24], are imperfect. Most methods are limited to inferred groups of orthologous sequences, and focus on estimating species trees rather than understanding the patterns of incongruence. Methods also exist for examining duplications using models of gene birth and loss [27, 28], though these can require dated trees. Finally, many methods used for phylogenetic reconstruction with genomic or transcriptomic data treat the coalescent process, gene duplication, and other sources of conflict as constant across the phylogeny (i.e., the same model parameters applied throughout) which becomes increasingly untenable with more extensive taxon sampling.

Transcriptomic and genomic datasets present a number of unique challenges for phylogenetic analyses in addition to gene tree and species tree conflict. Computational challenges often limit the amount of data or type of analyses that can be conducted (e.g., [1, 3, 6, 9]). Errors may be introduced at many stages throughout dataset construction, including during sequence assembly [29], during amino acid translation, and during homology inference. Problems with accurate homology inference in particular have forced dramatic reductions in the number of gene regions used in previous analyses [26]. Moreover, most existing phylogenetic analysis programs require homolog groups to be parsed into groups of orthologous sequences for analysis (but see [11, 24]). Recent methods that greatly improve homology (including orthology) assessment have been shown to increase the number of loci usable in downstream phylogenetic analyses [26]. By examining homologs directly, we can bypass the need to confidently infer orthologs and can more directly analyze gene families. This is increasingly important as more gene and whole genome duplications are identified. Although these improved homology assessment pipelines are highly promising, they come at the cost of magnifying the computational problems associated with analyzing large numbers of genes. Hence, it is important to develop phylogenomic analyses that can accommodate the enormous size of these datasets, work with partially-overlapping taxon sets across gene regions, and explicitly deal with conflict among sets of genes.

Although progress in reconciling gene tree conflict for estimating species trees continues, detailed examination of the potential causes of these patterns in phylogenomic datasets has largely been ignored. In this paper we explore the distribution of conflict, concordance, and gene duplications in transcriptomic and genomic datasets derived from two disparate taxonomic groups (19 species in the Apocrita clade of Hymenoptera, and 67 species in the angiosperm clade Caryophyllales) as case studies in characterizing the underlying gene tree conflict across a phylogeny.

Both of these datasets have presented challenges in constructing species trees that the volume of transcriptomic data was meant to overcome. The aculeate Hymenoptera are an extremely diverse group of tens of thousands of species that includes all ants, bees, and wasps, and hence encompasses the evolution of diverse social insect behaviors. The crown group Aculeata originated approximately 150 Ma [30] and is distributed globally. The early diverging lineages of this group have remained difficult to resolve, which has resulted in significant data collection efforts [5]. To complement this dataset, we also examined patterns of gene tree conflict within the Caryophyllales. The Caryophyllales are an ecophysiologically hyperdiverse clade with an estimated 11,510 species in 35 families (APG III; [31]), representing approximately 6 % of extant flowering plant species diversity. They have an estimated crown age of ca. 121-67 Ma [32–34], are distributed on all continents and in all terrestrial ecosystems, and exhibit extreme diversity in life history strategies. Despite recent plastid-based phylogenetic studies that have resolved a number of relationships, many important deep relationships, including key radiations, remain unresolved [35–41]. In addition, some lineages of Caryophyllales have experienced multiple rounds of genome duplication as well as many smaller-scale gene duplications [42], providing an excellent opportunity to explore patterns of gene and genome duplications in a large, relatively ancient angiosperm clade that has been well sampled phylogenomically.

## Methods

### Datasets

The aculeate Hymenoptera dataset includes 18 ingroup taxa (11 transcriptomes, 1 low-coverage genome, 6 annotated genomes) and the annotated genome of one nonaculeate hymenopteran outgroup taxon (*Nasonia vitripennis*). Peptide sequences from the Hymenoptera dataset were kindly provided by the authors of [5] or were downloaded from NCBI (NCBI bioproject 66515; http://www.hgsc.bcm.edu/arthropods/bumble-bee-genome-project; [43–48]). The Caryophyllales dataset includes transcriptomes of 67 Caryophyllales taxa and annotated genomes of 27 outgroups across eudicots, for a total dataset of 96 taxa; this dataset is described in more detail in [42]. Peptide sequences were used in both cases to reduce issues related to saturation.

Homolog groups for the Caryophyllales were identified from [42], while homolog groups for the Hymenoptera were identified from [26]. Here we briefly summarize the methods for homology inference. For both datasets, we conducted a Markov clustering procedure [49] followed by iterative multiple sequence alignment using MAFFT (v. 7.14) [50] and/or SATe (v. 2.2) [51], ML phylogenetic analysis with RAxML (v. 8.0.2) [52], trimming of spurious tips and deep paralogs, and realignment and re-estimation of the homolog group phylogeny. Spurious tips are defined as tips that have extremely long branch lengths, suggestive of errors in alignment or homology assignment. For Caryophyllales, the resulting homolog trees that contain at least 60 of the 67 ingroup taxa were used for subsequent analysis here. Similarly, homolog trees from the Hymenoptera dataset that contain at least 18 of the 19 taxa were included for analyses here. For both datasets, we conducted 100 bootstrap replicates in RAxML for each homolog group and extracted the rooted ingroup homolog clades from homolog trees for further analyses.

For the Hymenoptera dataset, we recovered 5,863 homolog groups that were used for conflict and concordance analyses. For phylogenetic analyses, we then used a 1-to-1 orthologs approach to identify 1,116 ortholog groups that contained at least 16 of the 19 total taxa [26]. For the Caryophyllales, we used a phylogenetic tree-based approach to homolog identification and processed the homolog groups into ortholog groups using the ‘rooted ingroups’ orthology inference procedure described in [26]. We recovered 10,960 homolog groups that each contained at least eight ingroup taxa. From this set of homologs, we identified 1,122 ortholog groups that contained at least 65 taxa. These orthologs were concatenated and used to construct a phylogeny and had an ortholog occupancy of 92.1 %. Two samples were removed from the original analyses because of potential contamination. Of the original 10,960 homolog groups, 4,550 contained at least 60 taxa and these were used for conflict and concordance analyses. For both groups, we used RAxML (v. 8.0.2) with the PROTCATWAG substitution model to estimate ML topologies, with each data matrix partitioned by gene region. We will refer to these comprehensive phylogenetic hypotheses as ‘species trees’ below. The inferred species trees for Hymenoptera and Caryophyllales are presented in Figs. 2 and 4, respectively. We note here that while the inference of species trees is not the focus of the present study, they nevertheless are useful for mapping results of gene tree congruence and conflict. We also note that the concatenation-based species trees employed here are identical to coalescent-based species trees estimated for these groups [5, 42], with the exception of one highly mobile taxon in Caryophyllales, *Sarcobatus*.

In order to quantify the differences among homologs, we summarized a number of statistics on each homolog including the average molecular substitution rates of each clade (the average distance from the ingroup root to the tips), the proportion of edges within a homolog tree that had a bootstrap value greater than 50 %, and the average bootstrap value.

### Identifying and mapping conflict and congruence

Directly comparing whole gene tree topologies for conflict/congruence is limited in that topologies can only be identical or non-identical; topologies that are non-identical may nevertheless share a high proportion of identical internal edges. Such whole-topology comparisons become increasingly uninteresting as taxon sampling (tree size) increases. A more informative comparison involves an examination of shared internal edges (bipartitions) across topologies. To examine conflict and concordance we first deconstructed each edge in each rooted ingroup homolog clade into bipartitions. For each node in each rooted ingroup homolog clade, we recorded the taxa included in the clade (toward the tips) and the taxa that were not included in the clade (toward the root). Because the input trees were rooted, we considered the bipartitions to be rooted, which allowed for more precise conflict identification.

**Fig. 1:**
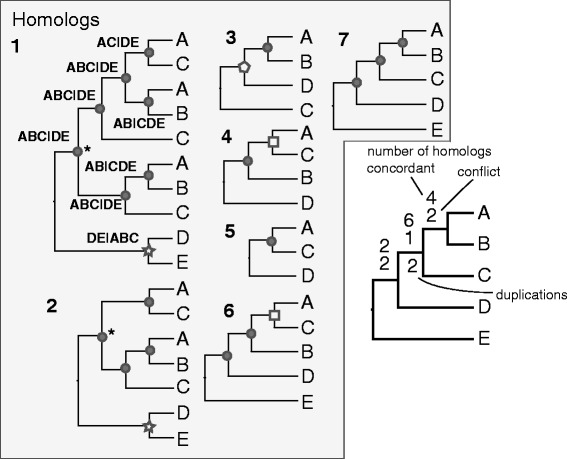
An example of mapping conflict, concordance, and gene duplication with gene trees (left) and on a species tree (right). The first gene tree has the bipartitions that are recognized noted at each internal node with ingroup on the left and outgroup on the right. The filled circles show clades that are concordant with the species tree, while open shapes correspond to nodes in conflict. The asterisks indicate recognized gene duplications (requiring at least two included taxa). The number of gene trees concordant, conflicting, and involved in gene duplications are noted on the species tree

Specifically, by establishing a root we allow for the identification of an ingroup clade and outgroup taxa set with respect to a node in reference tree. This allows us, for example, to distinguish between grades and clades through the unions and intersections of ingroup and outgroup taxon sets; this is not possible when working with unrooted trees. We also allowed bipartitions to contain gene duplications. So the bipartition (A,A,B) | (C,D,E) is recorded as (A,B) | (C,D,E) (see Fig. 1 for an example).

For each dataset, we deconstructed each rooted homolog ingroup tree and compiled the set of all unique bipartitions. By homolog ingroup tree we mean hypothesized clades within a homolog (i.e, gene tree). We also applied a bootstrap filter where edges with bootstrap values lower than 50 % were ignored. While the information was available to make comparisons across the entire set of unique bipartitions in each dataset, the combinatorics made this prohibitive. Instead we chose to summarize concordance and conflict in the bipartitions against the species tree topologies.

To summarize the concordance of the rooted ingroup homolog trees with the species tree topology, we started with the set of unique bipartitions. We then proceeded through the species tree, comparing each bipartition from each gene tree, recording whether the bipartition was concordant with or conflicted with each clade in the species tree. We then reported the number of homolog groups concordant or conflicting with the clade in the species tree. We considered a homolog tree bipartition (*h*) to be concordant with the species tree bipartition (*s*) if 1) the ingroup of *s* contains all of the ingroup of *h*, and 2) the outgroup of *s* contains all of the outgroup of *h*; if *h* is consistent with several *s*, *h* is mapped to the shallowest *s* (i.e. furthest from the root). We considered a bipartition *h* to be in conflict with *s* if 1) the ingroup of *h* contains any of the ingroup of *s*, 2) the ingroup of *h* contains any of the outgroup of *s*, and 3) the ingroup of *s* contains any of the outgroup of *h*.

To summarize the distribution of conflicting topologies, we binned all conflicting bipartitions into groups that were internally concordant. For each conflicting bipartition found with the above procedure, we conducted an all-by-all comparison to group bipartitions that make the same phylogenetic statement about the alternative resolution. We grouped bipartitions that were contained completely within another bipartition (e.g., as a result of reduced taxon sampling). This gave the number of homologs that supported alternative topologies at each node. Because a conflicting bipartition may be concordant with multiple alternative bipartitions, the cumulative sum of the homologs presented as alternatives may be larger than the total number of homolog trees.

### Information content measurement

[25] define the ‘internode certainty’ (ICA) metric that quantifies the degree of certainty for individual focal bipartitions (internal edges) by considering the frequency of all conflicting bipartitions. This is calculated for each internal edge, *i*, as:

(1) $$ ICA_{i}=1+\sum\limits_{n=1}^{b}P(X_{n})log_{b}[P(X_{n})]  $$

where *b* is the number of unique conflicting bipartitions (including the bipartition of interest, *i*) and *P*(*X*_*n*_) is the proportional frequency of bipartition *n* in the set of bipartitions being examined. ICA values near 0 indicate maximum conflict (i.e. conflicting bipartitions are of similar frequency), whereas values near 1 indicate strong certainty in the bipartition of interest. As originally implemented, this measure requires complete taxon overlap. Very few gene trees in the set of homologs contained all taxa, and many of these homolog trees contained gene duplications. However, the ICA measurement itself only requires the ability to calculate the frequency of conflicting and compatible bipartitions. We use the distribution of conflicting bipartitions as determined using the above procedure for calculating the ICA statistic on our species tree and homolog phylogenies. The nature of reduced taxon sampling reduces the accuracy of the ICA. To explore the behavior of the ICA when presented with gene trees with missing data we conducted simulations. We simulated 50 phylogenies under a pure birth process each with 50 taxa. For each tree, we rescaled the root to 10 and conducted 1000 coalescent tree simulations using COAL [53] to generate topological conflict with respect to each internal node in the original pure birth tree. We then randomly pruned each of the 1000 gene trees according to a set percentage of missing data. We conducted these simulations reducing the gene trees with 10 %, 20 %, and 30 % missing data. For the empirical datasets, we recorded the ICA statistic for each bipartition in the combined species tree. Alternative methods for calculating ICA with missing taxa, but without gene duplications, are described by Kobert et al. (http://dx.doi.org/10.1101/022053).

### Identifying and mapping duplications

To record gene duplications, we walked through each homolog tree in a postorder traversal (from tips to root). At each node, we recorded the ingroup descendant taxa. Then, we examined whether the children of the node contained multiple gene copies for at least two taxa. If this was the case, we recorded this node as containing a duplication. Because we required at least 2 taxa to be present, this method for duplicate identification loses power toward the tips of the species tree. This may be especially true for transcriptome data, or noisy data, where both duplicates may not be expressed or sequenced in all ingroup species. When a duplication was detected, the union of the descendant taxon sets was recorded at the focal node (to be compared when continuing to traverse down through the tree). A bootstrap filter of 50 % was applied as in the bipartition analyses. In this case, the focal node as well as the subtending left and right subtree nodes had to pass the bootstrap filter to be considered a duplication.

As with the identification of concordant and conflicting bipartitions, we mapped the number of gene duplications for each node in the species tree topology. While all duplications were recorded for each homolog tree, only those duplications that were congruent with the species tree were mapped.

All of the analyses discussed above are implemented in the open source java package phyparts (https://bitbucket.org/blackrim/phyparts).

### Coalescent gene tree simulations

Gene tree distributions and probabilities can be estimated based on a multi-species coalescent model [54]. In order to better determine whether the distribution of conflicting trees follows a pattern that could be explained by incomplete lineage sorting, we simulated gene trees on the species trees of Hymenoptera and Caryophylalles. In order to conduct these analyses, it is necessary to transform the species tree from branch lengths proportional to substitutions per site to branch lengths in coalescent time units (proportional to the product of population size *N*_*e*_ and mutation rate). Because we have no estimates of population size or mutation rate, and these are likely to have varied over the course of evolution for both groups, we transformed the trees to be ultrametric using treePL [55] and varied the root heights to be 10, 20 and 30. As branch lengths in these coalescent simulations reflect effective population size and mutation rate, if mutation rate is kept constant, these heights represent a broad range of effective population sizes. Under these conditions, deep coalescent events range from significantly frequent (as with 10) to relatively rare (as with 30). For each tree height, we generated 10,000 gene trees using COAL [53] and conducted the same bipartition analyses described above for the empirical datasets.

### Gene ontology association

For each of the homolog groups across both datasets, we associated gene ontology (GO) information. Specifically, we used blast with each alignment and annotated GO slim terms from *Arabadopsis* or *Drosophila*. For *Arabadopsis*, we used the genome annotations from TAIR [56]. For *Drosophila*, we used release FB2014_05 from FlyBase (flybase.org; [57]). GO terms are related to one another through a graph, and sequences may have from zero to many related GO terms. Because these terms can be nested, for each alignment we report the set of GO terms that were the most derived and contained within the set of GO slim terms.

## Results

### Hymenoptera results

**Fig. 2:**
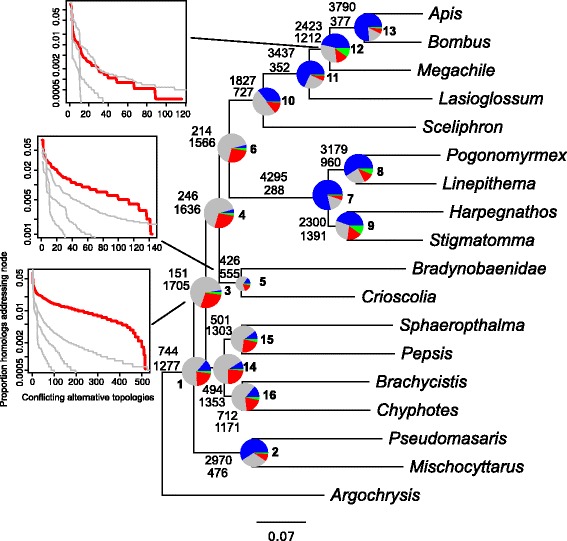
Combined ML (species tree) topology for Hymenoptera, with summary of conflicting and concordant homologs. For each branch, the top number indicates the number of homologs concordant with the species tree at that node, and the bottom number indicates the number of homologs in conflict with that clade in the species tree. The pie charts at each node present the proportion of homologs that support that clade (blue), the proportion that support the main alternative for that clade (green), the proportion that support the remaining alternatives (red), and the proportion that inform (conflict or support) this clade that have less than 50 % bootstrap support (grey). The histograms show, for three nodes, the proportion of the total homologs that support each conflicting alternative resolution for the clade in question, sorted from largest to smallest. Grey lines represent distributions of conflicting alternative resolutions based on coalescent simulations generated with three tree heights. The histograms for other nodes are presented in Additional file 2: Figure S5

**Fig. 3:**
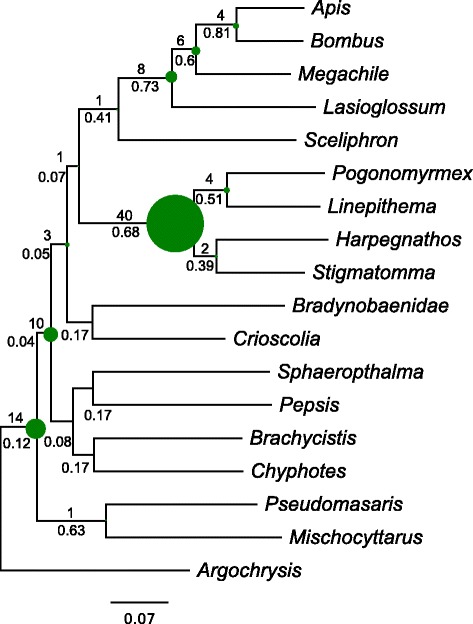
Inferred gene duplications and ICA values for Hymenoptera, mapped onto the same topology as in Fig. 2. The numbers above each branch are the number of gene duplications and numbers below each branch are the ICA values. The size of each circle is proportional to the number of duplications at that node

The species tree based on concatenated gene regions is discussed in [26] and is presented in Fig. 2. We calculated ICA scores on the species tree given the set of homolog trees. To explore the impact of missing taxa on the ICA measurements, we examined simulated data with missing taxa (Additional file 1: Figure S1). These results suggested that the ICA is generally conservative when data are missing in gene trees with increased uncertainty and noise as missing data increased. For the Hymenopteran results, ICA values ranged from 0.03 to 0.81 (Fig. 3). ICA values along the backbone were lower, ranging from 0.03 to 0.06, while ICA values in many of the nested clades were higher and ranged from 0.08 to 0.81. The highest values were found within Apoidea, with the clade uniting *Apis* and *Sceliphron* having the highest value (0.81). The original analyses of [5] recovered support values between 56 % and 100 % using the species tree methods PhyloNet [58] and STAR [59]; analyses by [26] recovered similar values for jackknife support. The ICA values calculated here are notably lower, indicating a great deal of underlying gene tree conflict.

For mapping the statistics presented below, we used the species tree and the 5,863 homolog group dataset. The numbers of bipartitions were 90,354 (no bootstrap filter), 65,758 (bootstrap filter = 20), 38,625 (bootstrap filter = 50), and 19,891 (bootstrap filter = 80). While these can be mapped to any topology, we calculated the concordance and conflict of the bipartition sets against the species tree topology under a bootstrap filter of 50 % (see Fig. 2).

The number of homolog groups concordant with each clade in the species tree varied significantly (see Fig. 2). Specifically, nodes 2, 7-9, and 11-13 each had more than 2,000 concordant homologs and as many as 4,295. The remaining nodes had fewer concordant homologs, ranging from 151 to 744. While no node had an alternative bipartition with higher numbers of concordant homologs compared to the bipartition in the species tree, nodes 3 and 4 both had alternative bipartitions with high numbers of supporting homologs relative to the supporting homologs in the species tree. The major alternative topology for node 3 included a clade with Vespidae wasps and *Argochrysis* but not ants, with 123 homologs supporting the alternative and 151 supporting the species tree resolution. Node 4 had 147 homologs supporting an alternative clade excluding ants and including wasps as compared to 246 homologs supporting the species tree resolution. These were contrasted with nodes such as node 7 supporting the monophyly of ants and 13 uniting *Apis* and *Bombus* with very little conflict as compared to the number of homologs supporting the species tree resolution.

The distribution of alternative topologies supported by conflicting homologs is presented in Additional file 2: Figure S5 with three cases presented in Fig. 2. Gene trees generated from coalescent simulations were plotted to compare distributions. The proportion of the total homologs that support each conflicting alternative resolution are sorted from largest to smallest with the grey lines representing distributions based on coalescent simulations. Distributions of conflicting homologs for nodes 2, 7, 8, 10, 11, 12, and 13 fell within the coalescent simulations while 5, 9, and 14-16 fell just outside of the coalescent distributions. Nodes 1, 3, 4, and 6 fell far outside and/or had different shapes to the distribution than the coalescent gene tree simulations. Concordant homologs had higher average bootstraps for every node and higher mean proportions of informative clades than discordant homologs (Additional file 3: Figure S2 and Additional file 4: Figure S3.

Homologs at nodes 3-6, 10-12, and 14-16 that were concordant with the species tree had average rates that were higher than homologs in conflict with the species tree at those nodes (Additional file 5: Figure S4), whereas concordant homologs at nodes 1, 8-9, and 13 had rates that were lower than those in conflict.

Using a bootstrap filter of 50 %, we detected 175 total gene duplications across 133 total homologs. Of these, 113 duplications representing 81 homologs could be mapped to clades in the concatenated species tree (Fig. 3). The edge with the most gene duplications subtended the ant clade (node 7). There were also a number of duplications found in the bees and Sphecidae wasps (nodes 10-13), and duplications were also found toward the root of the tree.

The distribution of GO terms for genes that were concordant or conflicting with each clade in the species tree topology did not differ. All distributions of GO terms are presented in Additional file 6: Figure S6.

### Caryophyllales results

**Fig. 4:**
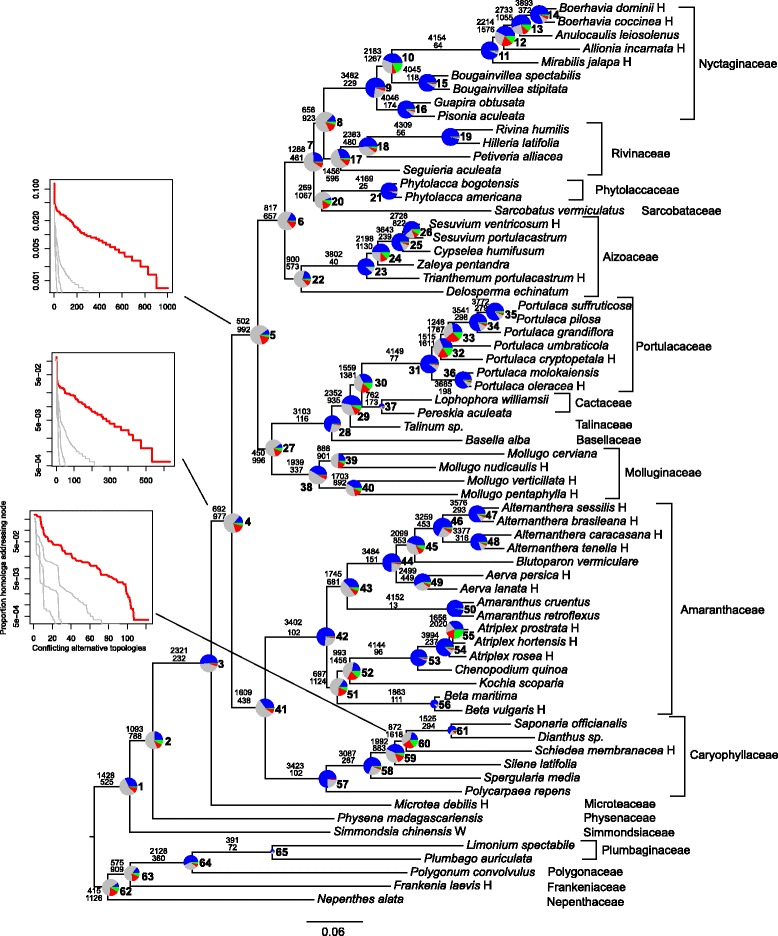
Combined ML (species tree) topology for Caryophyllales, with summary of conflicting and concordant homologs. Tree annotations follow Fig. 2. The histograms for other nodes are presented in Additional file 7: Figure S10

**Fig. 5:**
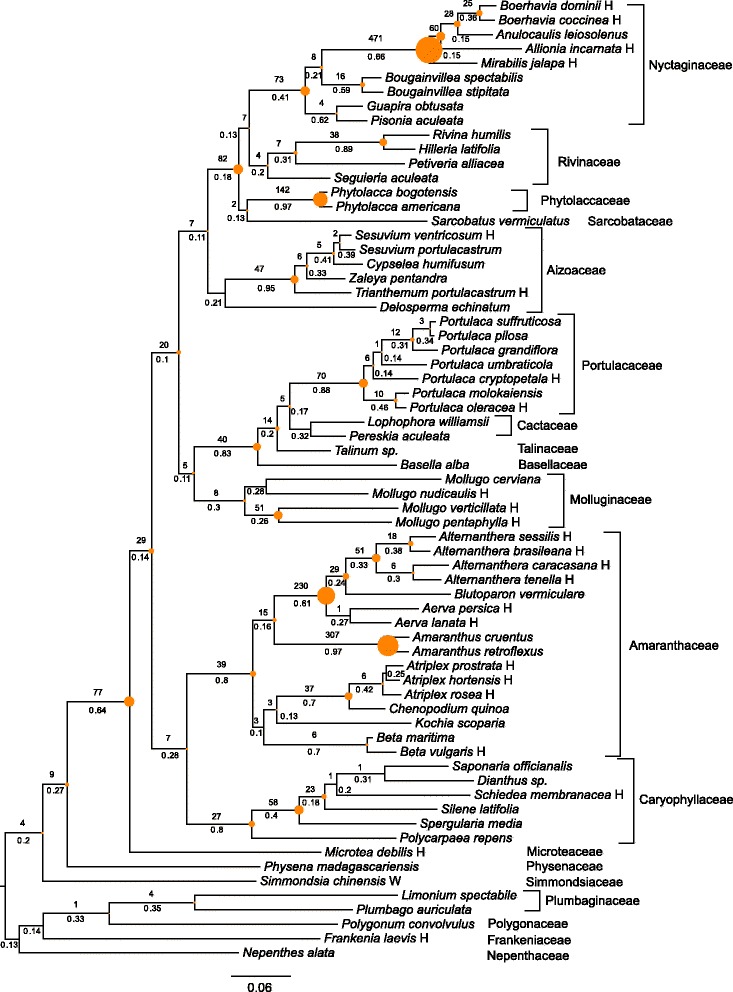
Inferred gene duplications and ICA values for Caryophyllales, mapped onto the same topology as in Fig. 4. The numbers above each branch are the number of gene duplications and numbers below each branch are the ICA values. The size of each circle is proportional to the number of duplications at that node

The species tree based on concatenated gene regions was discussed in [42] and is presented in Fig. 4. The bootstrap support was between 88 % and 100 % across the tree, but we found a large variation in ICA values, ranging from 0.08 to 0.97 (Fig. 5).

For example, the placement of *Sarcobatus* had 89 % bootstrap support but a 0.13 ICA. Values along the backbone ranged from 0.62 for the node separating Microteaceae from remaining core Caryophyllales to 0.12, 0.08, and 0.10 among other backbone nodes. Within major clades, values varied greatly. For example, in Amaranthaceae values were as high as 0.97 and as low as 0.10.

We used the species tree described above and the 4,550 homolog groups that contained at least 60 taxa to calculate the bipartition information (Fig. 4). The total number of bipartitions was as follows: 336,018 (no bootstrap filter), 287,971 (bootstrap filter = 20 %), 205,498 (bootstrap filter = 50 %), and 124,020 (bootstrap filter = 80 %). As with Hymenoptera, we calculated the concordance and conflict of the bipartition sets to the species tree topology using a bootstrap filter of 50 % (Fig. 4).

The number of concordant and conflicting gene regions varied greatly across the species tree. After the split from *Microtea*, the number of supporting homologs for the three backbone nodes of core Caryophyllales ranged from 502-817 and the number of conflicting homologs for the same nodes ranged from 657-992. These three backbone nodes, along with the split between Phytolaccaceae and Nyctaginaceae and the split between Molluginaceae and Portulacaceae+Cactaceae+Talinaceae+Basellaceae, had the lowest numbers of total informative homologs (i.e., concordant+conflicting homologs). The highest numbers of informative homologs were found nested within Amaranthaceae, Portulacaceae, Aizoaceae, Phytolaccaceae, and Nyctaginaceae. The distribution of genes concordant with alternative topologies is presented in Additional file 7: Figure S10, with specific distributions highlighted in Fig. 4. The proportion of the total homologs that support each conflicting alternative resolution are sorted from largest to smallest with the grey lines representing distributions based on coalescent simulations. With the exception of node 20, i.e., the placement of *Sarcobatus*, no alternative topology had a higher number of concordant genes than the bipartition found in the species tree from concatenated analyses. The alternative placement of *Sarcobatus* is supported by 340 homologs and places *Phytolacca* species sister to the Nyctaginaceae. Distributions of conflicting homologs for all nodes except 1,2,4-6,8,20,25-27,37,38,43,51,55,59,61-65 fell within or near the coalescent simulations (Additional file 7: Figure S10).

The average bootstrap values for each homolog concordant with the species tree were higher than conflicting homologs except for nodes 19-21, 26, and 50. The proportion of informative clades for gene trees of each homolog was higher for homologs concordant with the species tree for every node except nodes 26 and 50. The average rate of each homolog was higher for concordant homologs for nodes 4-8, 10, 30, 38, 59, and 65, and lower for concordant homologs for nodes 9, 15, 21, 23, 28, 31, 40, 51, 53, and 56. For details on each of these results, see Additional file 8: Figures S7, Additional file 9: Figure S8 and Additional file 10: Figure S9.

A much higher number of gene duplications (including repeated duplications) was detected in the Caryophyllales than in the Hymenoptera. Using a bootstrap filter of 50 %, we found 2,390 duplications across 1,532 homologs, resulting in an average of 0.5 duplications per homolog tree. Of these, 2,359 duplications representing 1,515 homologs could be mapped to clades in the concatenated species tree (Fig. 5). The most gene duplications were found within the Nyctaginaceae and Amaranthaceae. There were also high numbers of duplications found at other clades within the Sarcobatus+Phytolaccaceae+Nyctaginaceae clade, the base of Portulacaceae and at the split between Microteaceae and the core Caryophyllales.

As with Hymenoptera, the distribution of GO terms for genes that were concordant or conflicting with each clade in the species tree topology did not differ substantially. All distributions of GO terms are presented in the Additional file 11: Figure S11

## Discussion

In both the Hymenoptera and Caryophyllales datasets, our analyses reveal a significant amount of underlying gene tree conflict at most internal nodes. This is not surprising considering that the use of general transcriptomic and genomic datasets does not filter for a single phylogenetic signal and instead generates data broadly across the genome. Additionally, in both cases, phylogenetic relationships have been difficult to resolve in previous analyses. This phylogenetic recalcitrance has been assumed to be due to either conflicting and/or lack of phylogenetic signal, and our analyses suggest that both of these problems are present.

For more traditional datasets, the bootstrap, jackknife, or Bayesian posterior distributions are used to better understand the uncertainty associated with edges in the phylogeny. For transcriptomic and genomic datasets, these measures can be less informative (but see the jackknife approach in [26]). For example, only a small minority of nodes show lower than 100 % bootstrap or jackknife support in both the Hymneoptera and the Caryophyllales examples [26, 42]. The ICA score was developed to provide more information about the distribution of conflict at internal edges for large gene tree datasets [25], and it is extended here to accommodate partially overlapping taxa among loci. This score is not directly comparable to the bootstrap or jackknife. However, the ICA scores may be more relevant for phylogenomic datasets as they better reflect the underlying variation for both the Caryophyllales and Hymenoptera datasets. In both groups, all nodes had lower than 1.0 ICA value in the species trees, reflecting the presence of conflict at each node. However, as with any single edge-based metric, it can be hard to break down precisely what is causing the conflict at a particular edge and the distribution of alternative topologies and their frequencies.

Although the ICA metric addresses a relevant question for the datasets examined here, there is more information still to be ascertained from general concordance and conflict information. For example, the majority of homologs for most of the backbone nodes lacked phylogenetic signal (grey areas of pie charts in Figs. 2 and 4). This fact has surely contributed to the difficulty in resolving these clades, giving the impetus for the generation of these data. These backbone nodes also tend to have a very noisy distribution of conflicting genes. This contrasts with some of the well-supported nested clades, like *Atriplex* nodes 54 and 55, where alternative topologies that have high support are simply the rearrangement of closely related taxa. Both of these types of conflict are masked by simple bootstrap analyses.

While determining the numbers of bipartitions that are concordant or conflicting with a focal bipartition is helpful, further characterization of the set of homologs that are alternatively conflicting or concordant with a given node may also help diagnose issues within these datasets. We summarized three different aspects of the genes supporting or conflicting with each topology including the average molecular substitution rates of each clade (the average distance from the ingroup root to the tips), the proportion of edges within a homolog tree that had a bootstrap value greater than 50 %, and the average bootstrap value. Each of these had more homogeneous patterns in the Hymenoptera dataset than in the Caryophylalles dataset. The Hymenoptera dataset had a higher proportion of concordant homologs with informative gene trees at each node in the species tree, measured by individual homolog trees with nodes >50 % bootstrap support and higher average bootstrap value per homolog tree. For Caryophyllales, fewer nodes were characterized by having a majority of informative homologs. The average molecular evolutionary rates of the concordant and conflicting genes also displayed more pronounced patterns in the Hymenoptera dataset than the Caryophyllales dataset, likely reflecting the fact that compared to Hymenoptera, the Caryophyllales dataset is more heterogeneous in both node ages and information content.

It can be tempting to use some of these measures for filtering of gene regions for phylogenetic or other evolutionary analyses. Average bootstrap values have been suggested as a measure on which genes can be filtered for concatenated analyses [23]. Although Hymenoptera show some general patterns for these measures, the lack of an overall pattern for the Caryophyllales suggests that average bootstrap values may not always be an optimal method for filtering gene trees and to minimize conflicting signal. Moreover, there were numerous examples where gene trees with high information content conflicted strongly with other gene trees at the same internal edge. By filtering for homologs with higher *average* bootstrap values, homologs may be included in phylogenetic analyses that increase conflict across the tree. The sensitivity of gene tree/species tree methods to this conflict, combined with spurious phylogenetic resolution in gene trees with low information, has been the impetus for the development of binning methods [19, 22]. Our study is different in that our major aim is to dissect these conflicts rather than to estimate a single species tree.

Genuine phylogenetic conflict may be caused by a number of different biological processes, including hidden paralogy, ILS, horizontal gene transfer, and hybridization [13]. Given our current understanding of the patterns of these processes, it cannot be rejected that the species trees presented here have many nodes with low phylogenetic support due to both ILS and a lack of phylogenetic signal. The distribution of conflicting homologs for most nodes falls within distributions of gene trees generated from simulations on a neutral coalescent model under a variety of effective population sizes (Figs. 2 and 4; Additional file 2: Figure S5 and Additional file 7: Figure S10). For both datasets, this may be the result of rapid radiations, where lineage diversification is rapid enough that 1) few substitutions occur to register branching events and 2) not enough time elapses for polymorphisms to sort within a lineage. Gene and genome duplications that have occurred along these short branches further reduce the phylogenetic signal at these nodes. Nevertheless, there may be identifiability problems with comparisons to coalescent gene trees in that there are multiple processes beyond ILS that can contribute to gene tree distributions that mimic those expected from neutral coalescent processes. (e.g., introgression, see [60]). For example, two nodes within the Caryophyllalles that have high support for alternative topologies involve (1) closely related species of *Atriplex* and (2) the relationship of *Sarcobatus* and the Phytolaccaceae. This conflict may result from ILS at the point of speciation, but may also result from hybridization events.

Additionally, there are nodes in both trees that fall outside of these simulations and exhibit significant variation in the corresponding tree height (e.g., effective population size and mutation rate). These results may be due to very small effective population size, variation in population size or mutation rate through time, other changes in population structure, significant selection, or alternative sources of conflict, all of which would be violations of gene tree and species tree methods that assume a neutral coalescent. Although we acknowledge significant selection as a possible source of conflict, it is unlikely to be playing a significant role in generating conflicting signal. While different types of selection (purifying, positive, etc.) have undoubtedly occurred in many of these genes over time, the relative uniformity in gene ontology information between conflicting and concordant genes is more consistent with a rapid radiation. It is possible that if selection were playing a major role in generating conflict, we may expect a stronger signal of support or conflict in particular GO categories. This expectation needs to be explored in more depth. Nevertheless, no major difference is noted in the distribution of categories between any nodes, conflicting or concordant.

While the expected patterns of conflict and concordance can be explored for neutral coalescence and relatively simple models [53, 61], more investigation into the expected patterns of concordance and conflict under different processes and parameterizations is necessary. Additionally, further work is required to determine the sensitivity to detecting deviations from the neutral coalescent and the robustness of violations of the coalescent model in gene tree/species tree reconstruction. Transcriptome-based phylogenies involving distantly related species are likely to cover wide ranges of variation in effective population size, selection strengths, generation time, and substitution rate. Many processes will surely play at least a partial role at each clade and it will remain difficult to distinguish the relative contributions of these processes without more thorough examinations into the expected patterns associated with each.

One distinction of the approach taken here, after the suggestions of [26], is the examination of homologs instead of orthologs alone. As a result, our analyses provide power for resolving the phylogenetic location of gene duplication events involving multiple species. We use a counting method to identify duplications. While more sophisticated methods using models of gene birth and loss exist [27, 28], they may require divergence time estimates which can be further complicated by rate heterogeneity. The counting approach tends to be more conservative, however, and so we may underestimate the number of duplications [27]. Because we are interested in distribution of gene duplications instead of genome duplications, we applied a local bootstrap filter on individual bipartitions, instead of a global bootstrap filter that applied on the average bootstrap values across a homolog as in [42]. In addition, we only mapped gene duplications in bipartitions that are in concordance with the species tree. We found more gene duplications were detected within the plant lineage (Caryophyllales) than in Hymnenoptera. In addition to more extensive sampling within Caryophyllales, this result is likely due to repeated ancient genome duplications, particularly within the phytolaccoid clade and Amaranthaceae [42]. Additional gene duplication events may have occurred along the backbone of the Caryophyllales tree, although such deeper duplications are more difficult to characterize due to the smaller number of informative genes and the greater phylogenetic uncertainty surrounding these internal edges. Within the Hymenoptera dataset, the highest number of gene duplications was detected at the base of the ant clade and within the bee clade.

## Conclusion

Characterizing conflict and concordance among gene trees is a necessary prerequisite for evolutionary analysis of genomic-scale data. In turn, adequately accounting for the underlying conflict among loci within transcriptomic and genomic datasets is essential for estimating species trees. However, we emphasize that much more information of evolutionary interest is present within these large datasets beyond that necessary to infer phylogeny alone. We have demonstrated an approach that can serve as a useful first step in exploring the heterogeneous processes involved in genome evolution, including ILS, hybridization, and gene and genome duplications. Nevertheless, the development of additional methods will be necessary to tease apart the interplay of such processes at any particular region of the Tree of Life. The increasing ease with which genomic and transcriptomic data can be generated make the development of such methods pressingly critical. Without exploring the underlying processes and patterns that underlie these data, significant evolutionary events are ignored.

## Availability of supporting data

The datasets supporting the results of this article are available in the DataDryad repository, http://dx.doi.org/10.5061/dryad.5b568.

## Additional files

Additional file 1
**Figure S1.** Simulation results for ICA measure comparing estimates from complete datasets and datasets with varying amounts of missing data.

Additional file 2
**Figure S5.** The proportion of the total homologs in the Hymenoptera dataset that support each conflicting alternative resolution, sorted from largest to smallest. Grey lines represent distributions based on coalescent simulations. Node numbers correspond to those in Fig. 2 in the main text.

Additional file 3
**Figure S2.** Density plots of the average bootstrap values for homologs in the Hymenoptera dataset that are concordant with the node in question (blue) and those that are in conflict (red). Node numbers correspond to those in Fig. 2 in the main text.

Additional file 4
**Figure S3.** Density plots of the proportion of nodes that have a bootstrap greater than 50 for homologs in the Hymenoptera dataset that are concordant with the node in question (blue) and those that are in conflict (red). Node numbers correspond to those in Fig. 2 in the main text.

Additional file 5
**Figure S4.** Density plots of the average root to tip rates of molecular evolution for homologsin the Hymenoptera dataset that are concordant with the node in question (blue) and those that are in conflict (red). Node numbers correspond to those in Fig. 2 in the main text.

Additional file 6
**Figure S6.** The distribution of gene ontologies for homologs in the Hymenoptera dataset that are concordant with the node in question and those that are in conflict with the node in question. Node numbers correspond to those in Fig. 2 in the main text.

Additional file 7
**Figure S10.** The proportion of the total homologs in the Caryophyllales dataset that support each conflicting alternative resolution, sorted from largest to smallest. Grey lines represent distributions based on coalescent simulations. Node numbers correspond to those in Fig. 4 in the main text.

Additional file 8
**Figure S7.** Density plots of the average bootstrap values for homologs in the Caryophyllales dataset that are concordant with the node in question (blue) and those that are in conflict (red). Node numbers correspond to those in Fig. 4 in the main text.

Additional file 9
**Figure S8.** Density plots of the proportion of nodes that have a bootstrap greater than 50 for homologs in the Caryophyllales dataset that are concordant with the node in question (blue) and those that are in conflict (red). Node numbers correspond to those in Fig. 4 in the main text.

Additional file 10
**Figure S9.** Density plots of the average root to tip rates of molecular evolution for homologs in the Caryophyllales dataset that are concordant with the node in question (blue) and those that are in conflict (red). Node numbers correspond to those in Fig. 4 in the main text.

Additional file 11
**Figure S11.** The distribution of gene ontologies for homologs in the Caryophyllales dataset that are concordant with the node in question and those that are in conflict with the node in question. Node numbers correspond to those in Fig. 4 in the main text.
