# Supplementary figures and images for: Analysis of phylogenomic datasets reveals conflict, concordance, and gene duplications with examples from animals and plants
Source: BMC Evol Biol. 2015 Aug 5;15:150. doi: 10.1186/s12862-015-0423-0 (PMC4524127; doi:10.1186/s12862-015-0423-0)

10% missing data

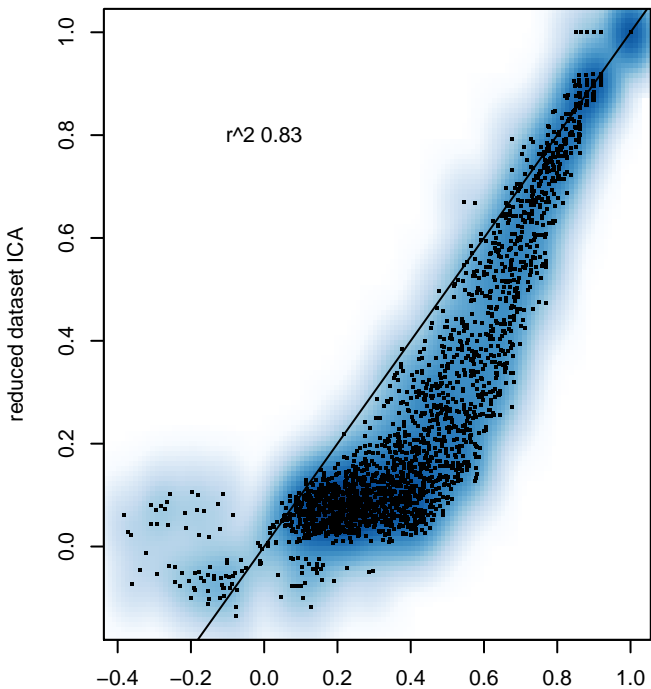

20% missing data

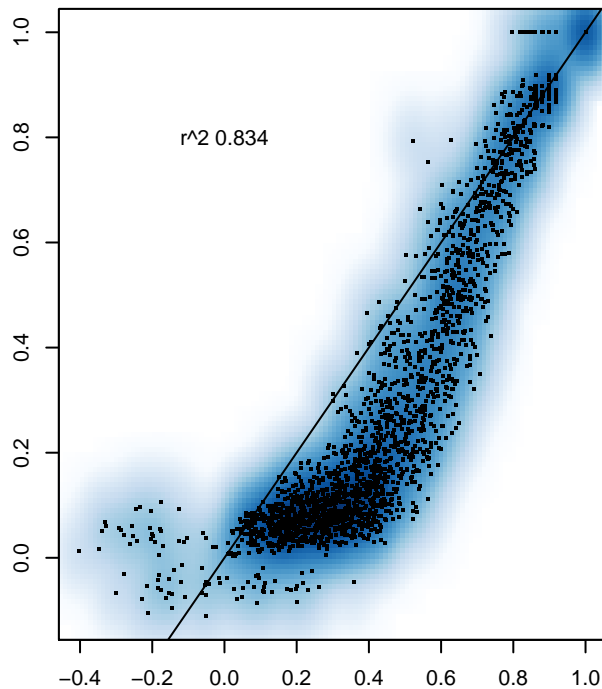

30% missing data

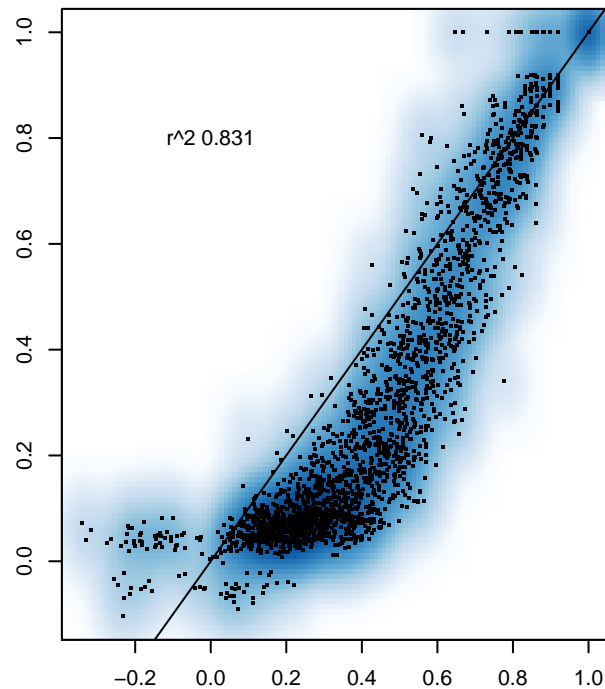

full dataset ICA

Supplement: Additional file 1 — Figure S1. Simulation results for ICA measure comparing estimates from complete datasets and datasets with varying amounts of missing data. [file 12862_2015_423_MOESM1_ESM.pdf]

Proportion homologs addressing node

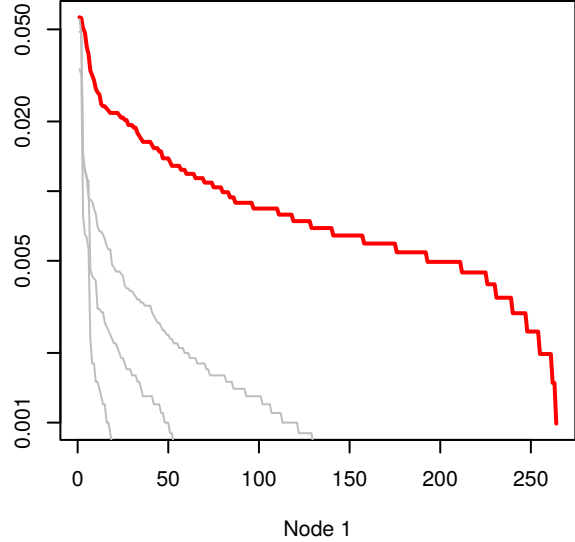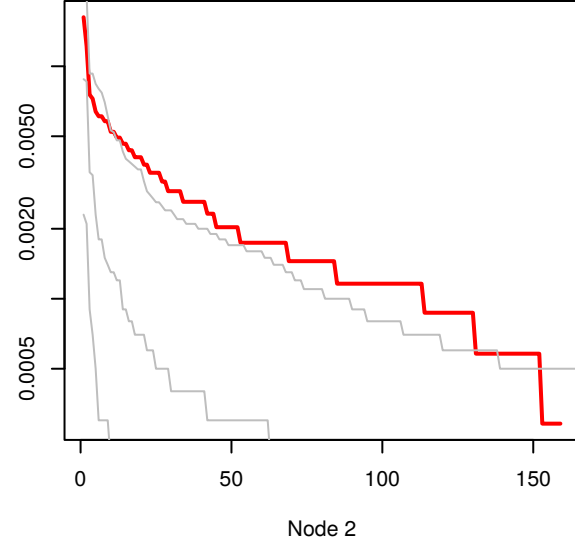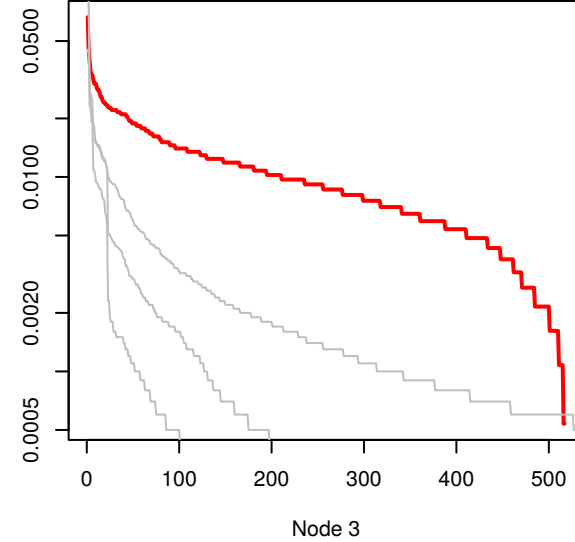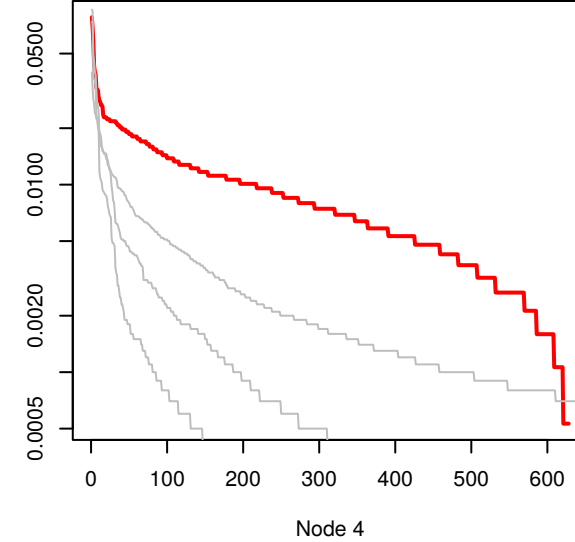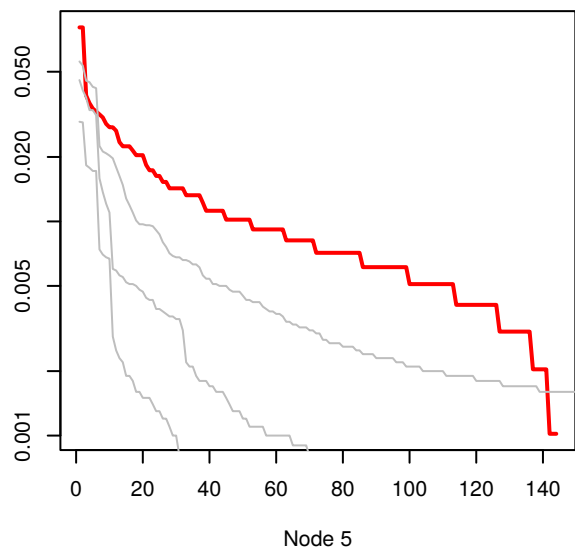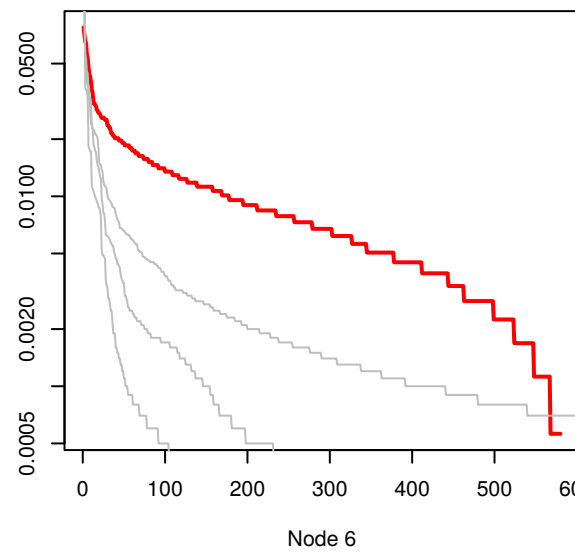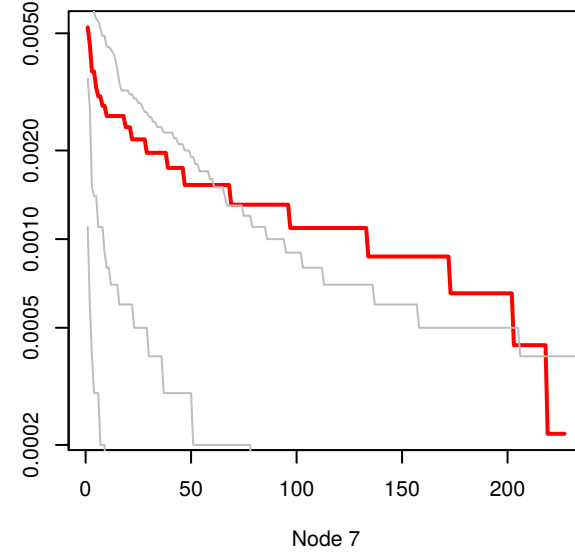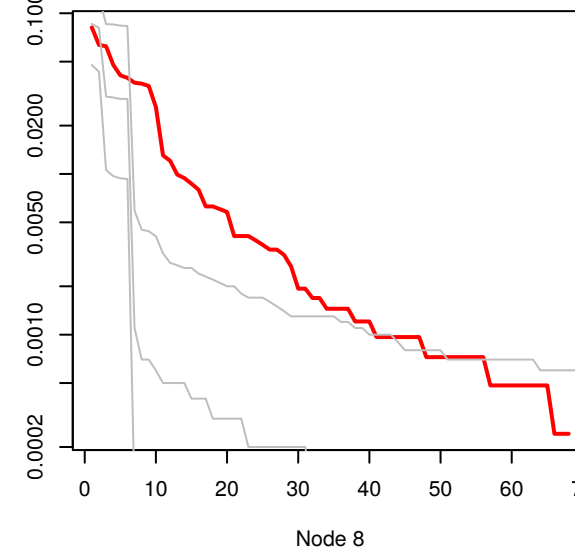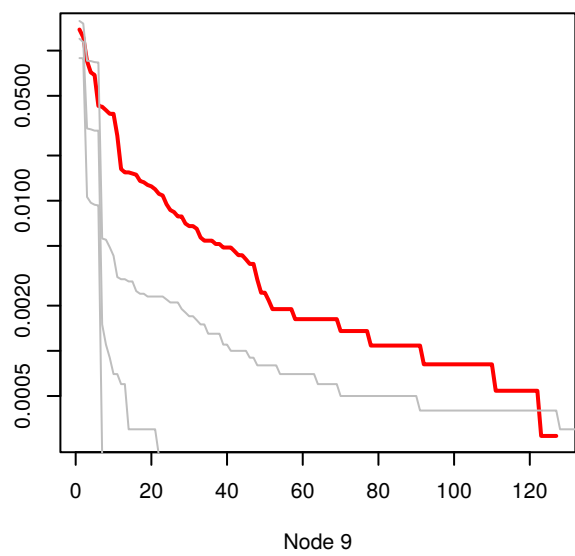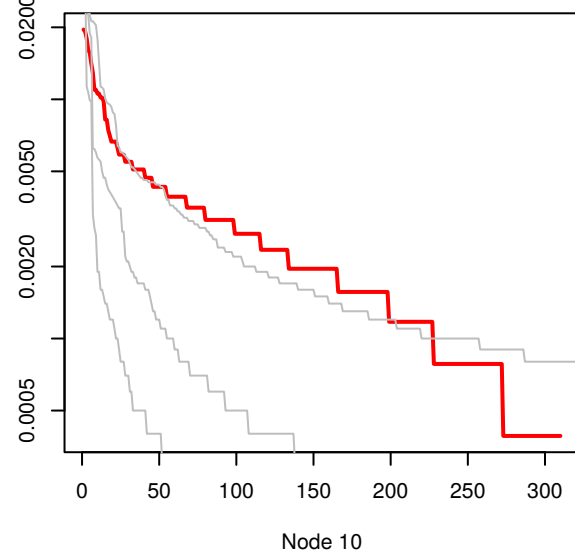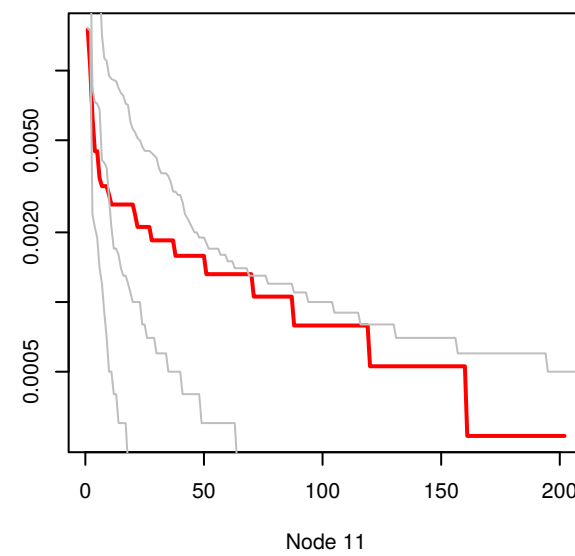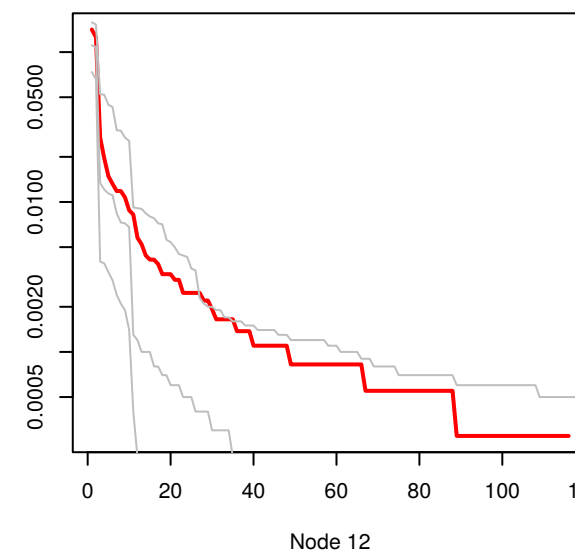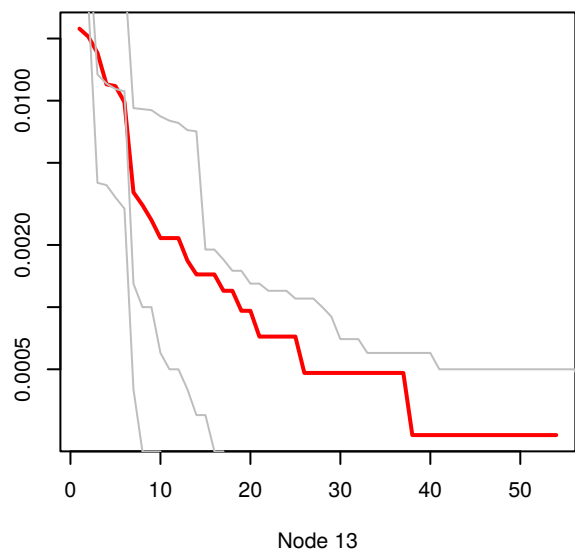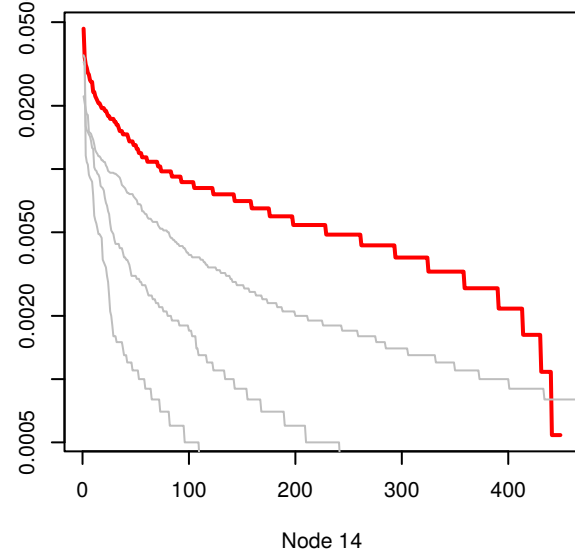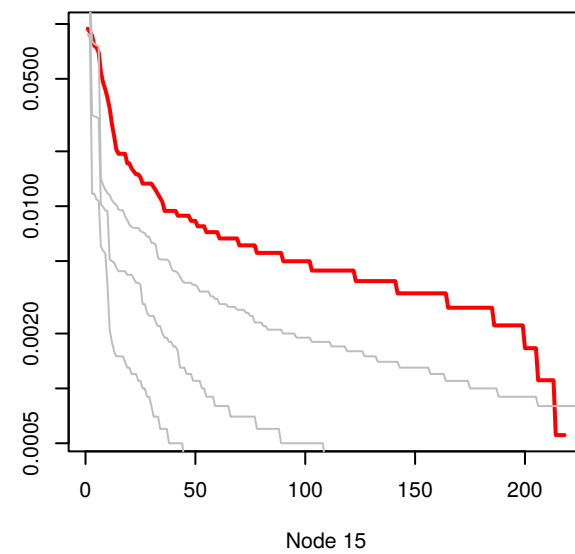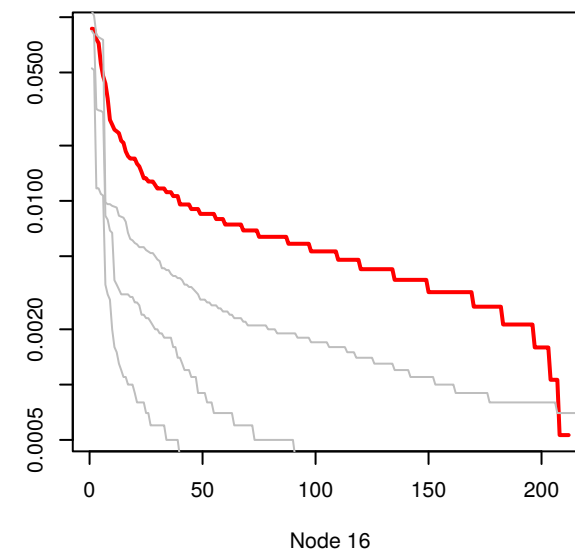

Conflicting alternative topologies

Supplement: Additional file 2 — Figure S5. The proportion of the total homologs in the Hymenoptera dataset that support each conflicting alternative resolution, sorted from largest to smallest. Grey lines represent distributions based on coalescent simulations. Node numbers correspond to those in Fig. 2 in the main text. [file 12862_2015_423_MOESM2_ESM.pdf]

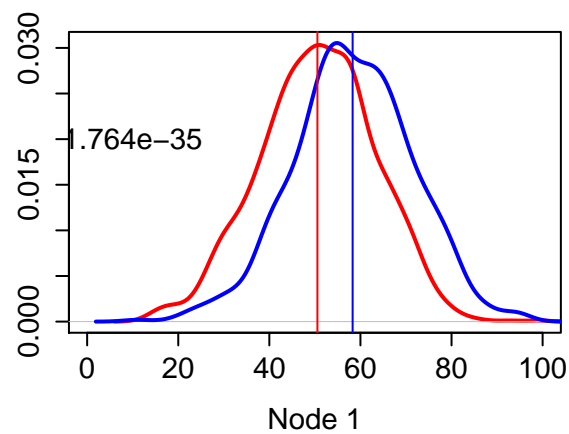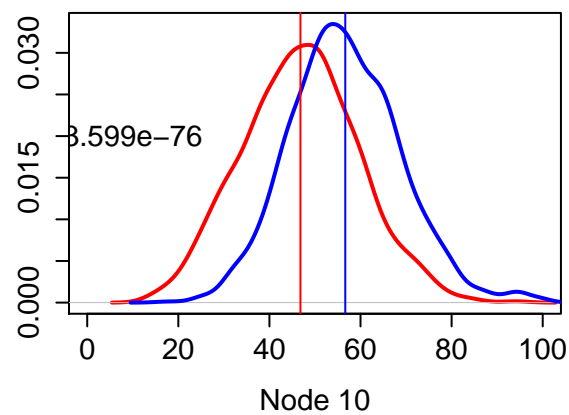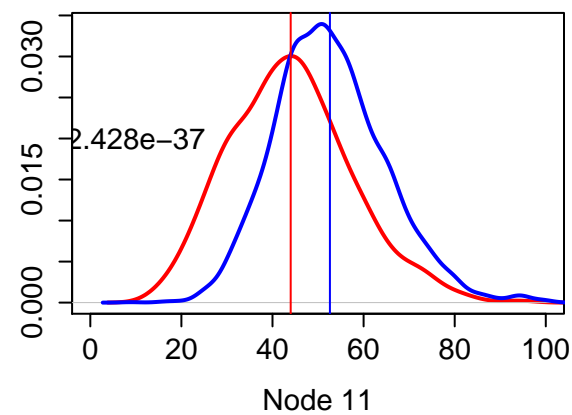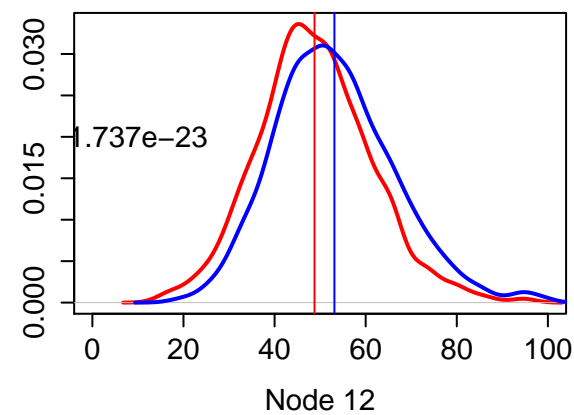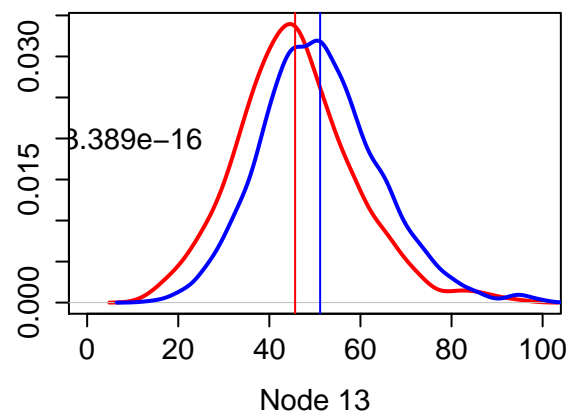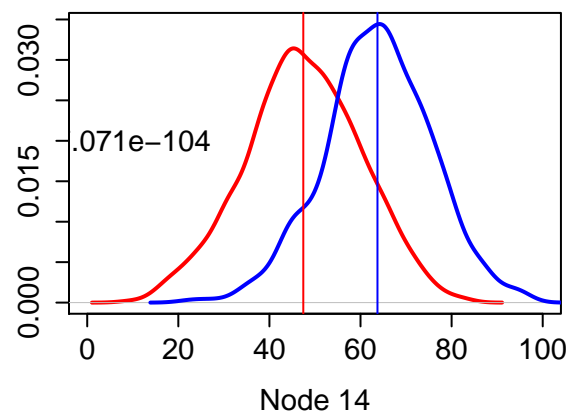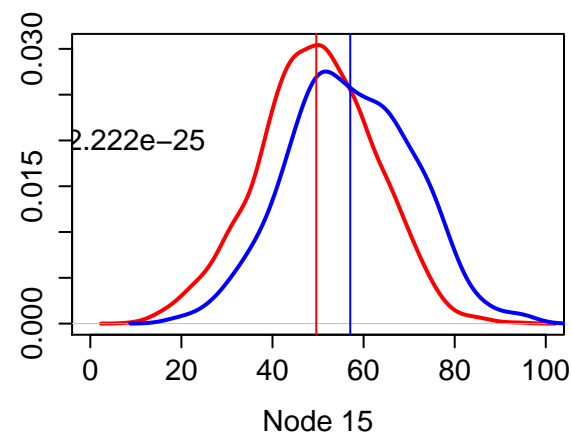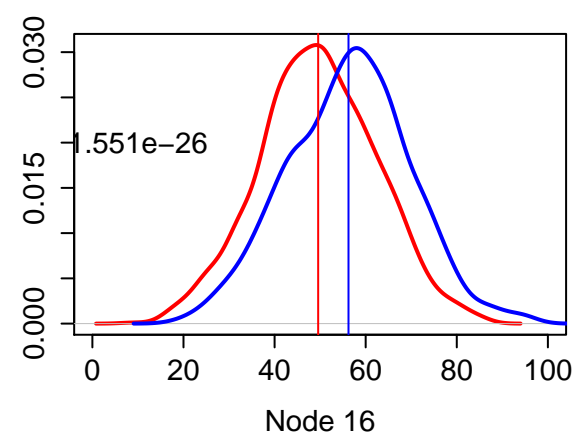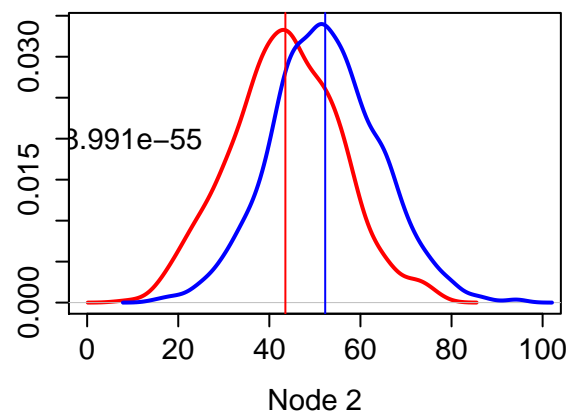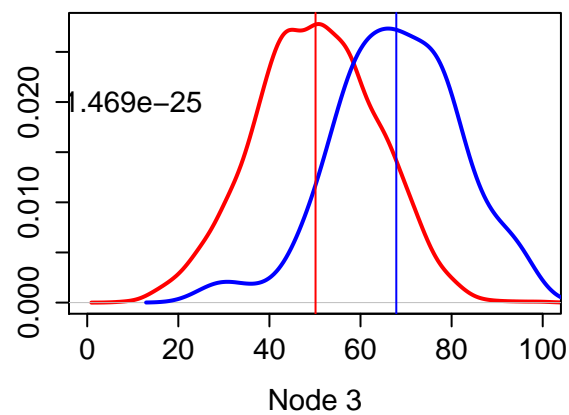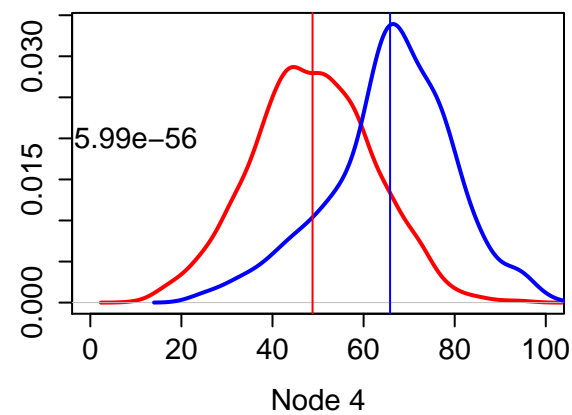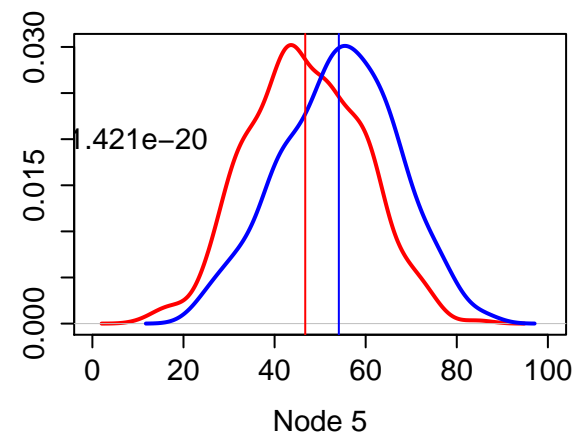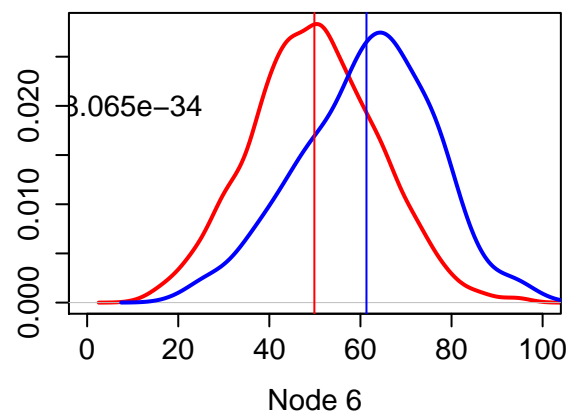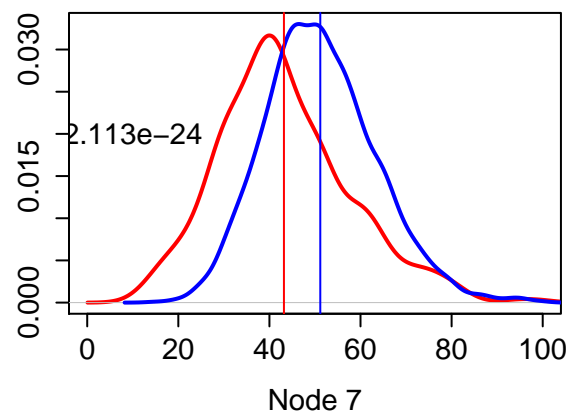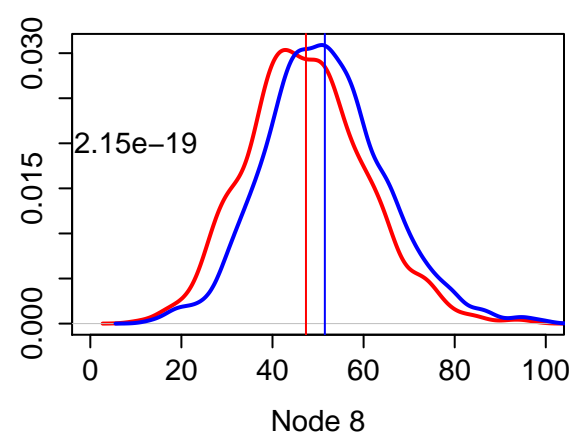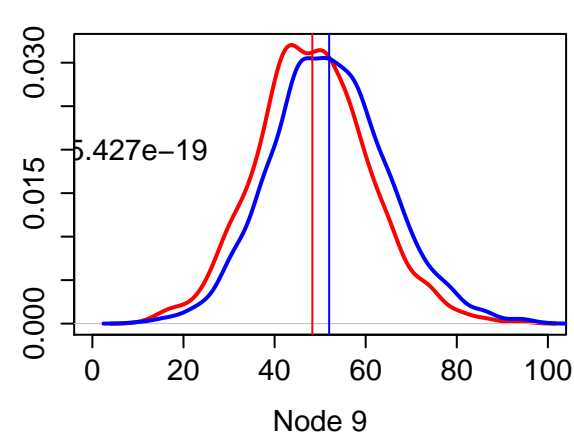

Supplement: Additional file 3 — Figure S2. Density plots of the average bootstrap values for homologs in the Hymenoptera dataset that are concordant with the node in question (blue) and those that are in conflict (red). Node numbers correspond to those in Fig. 2 in the main text. [file 12862_2015_423_MOESM3_ESM.pdf]

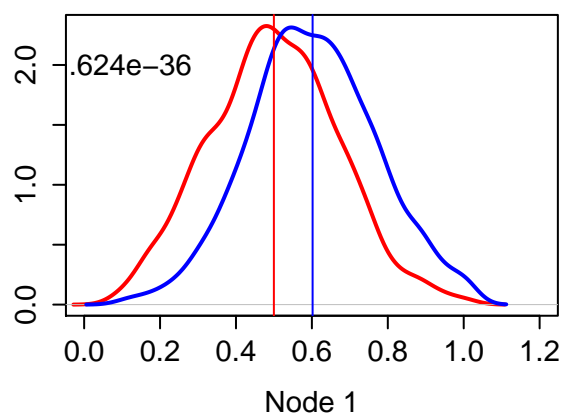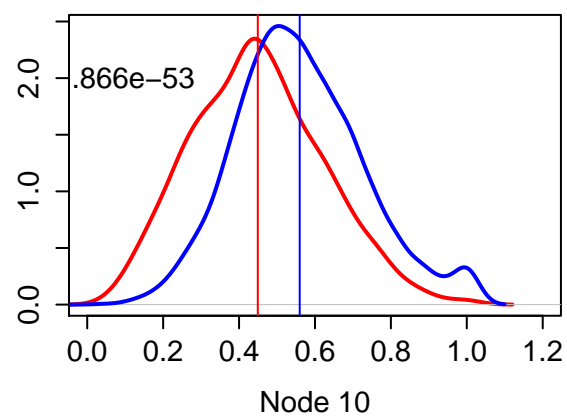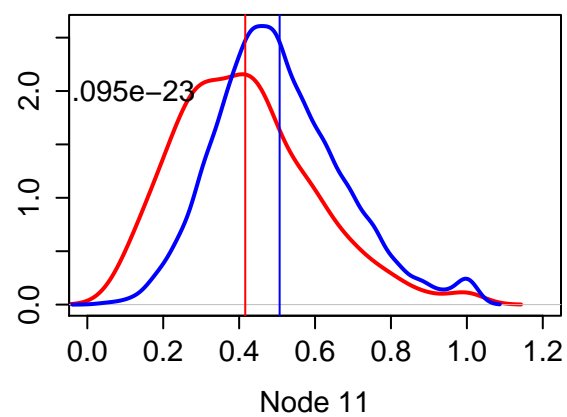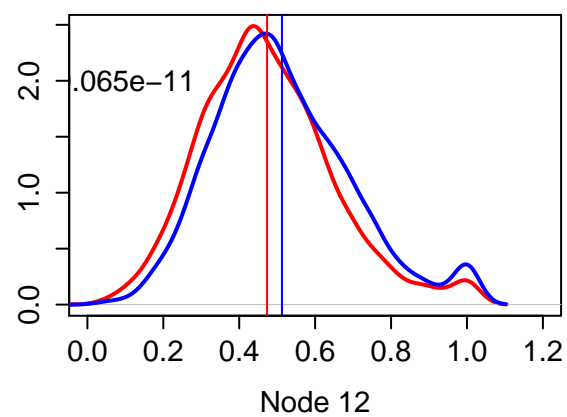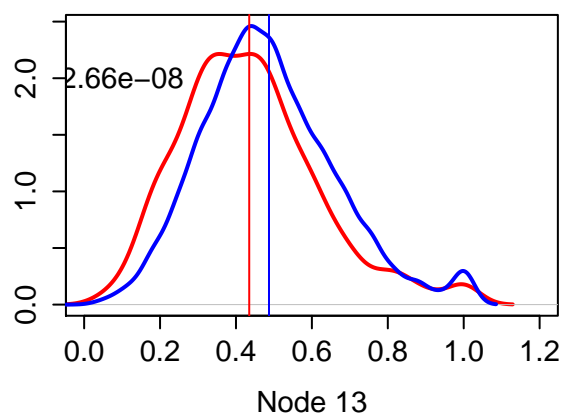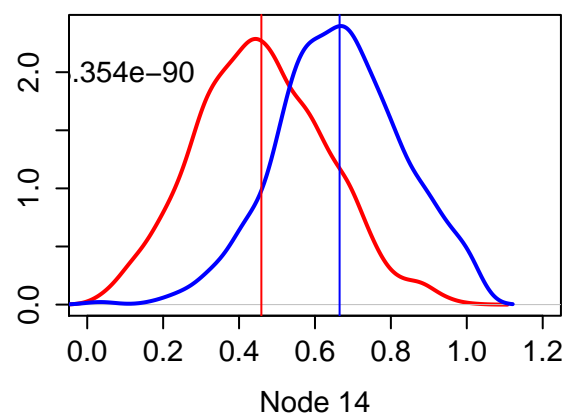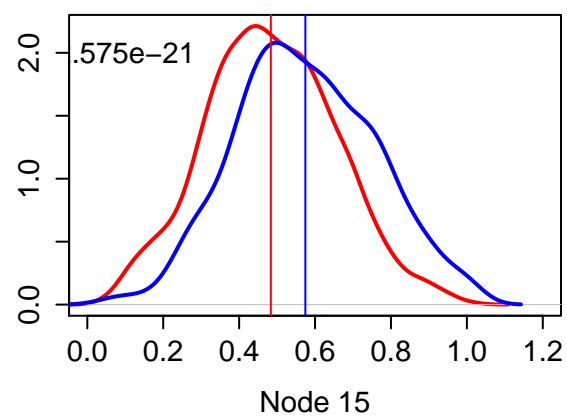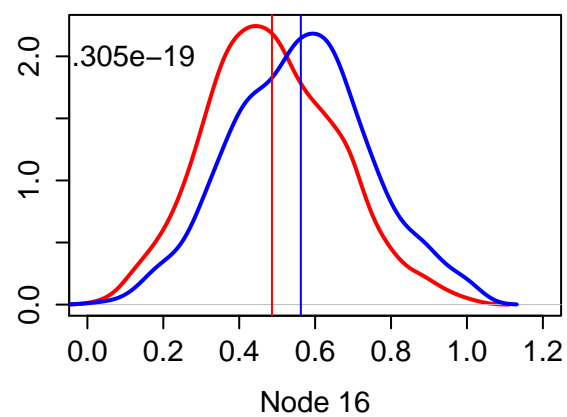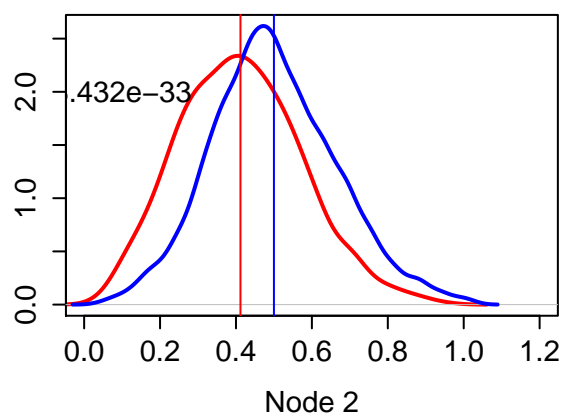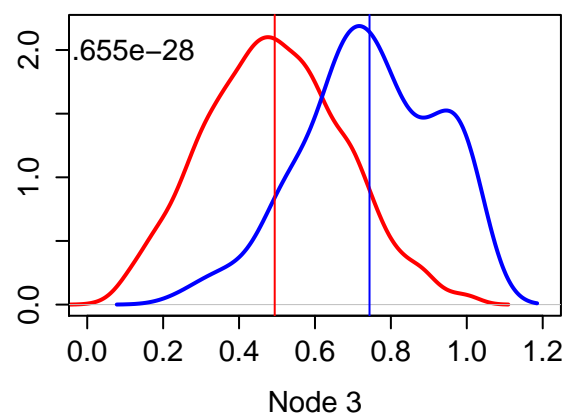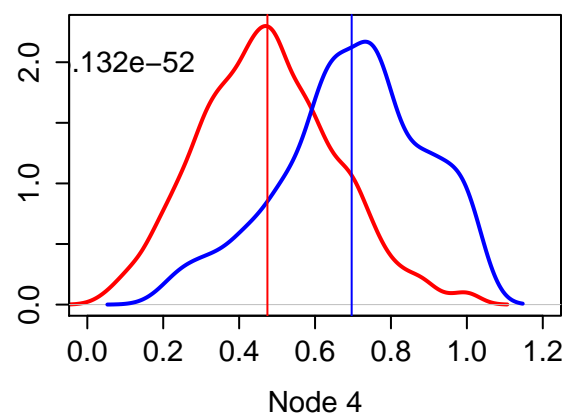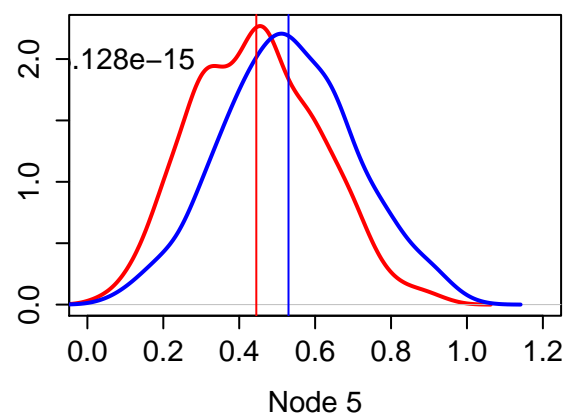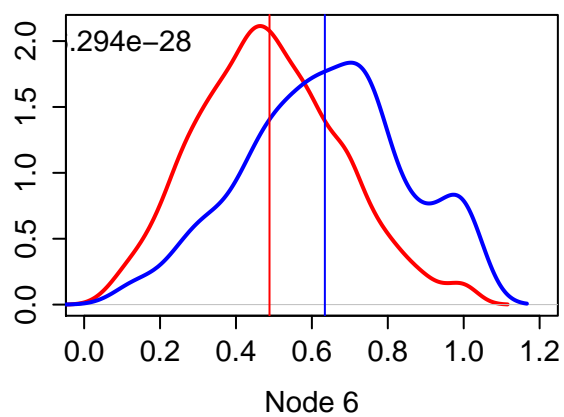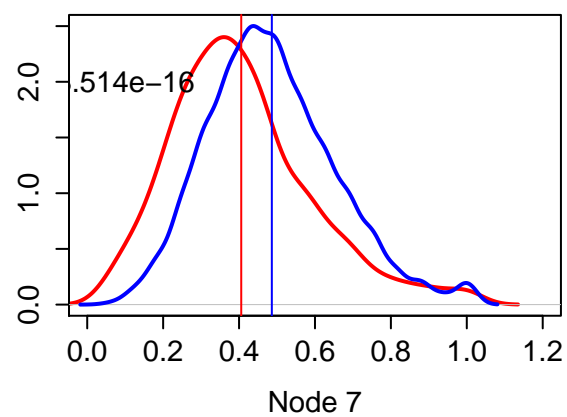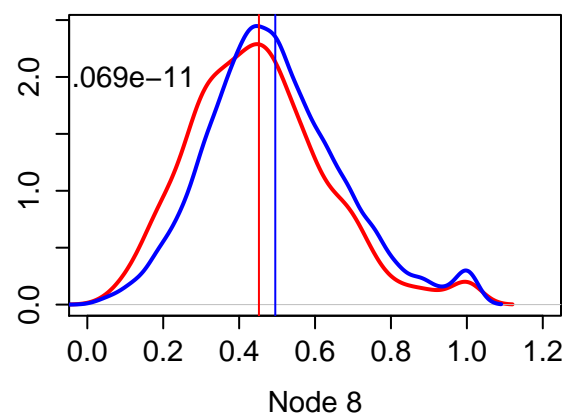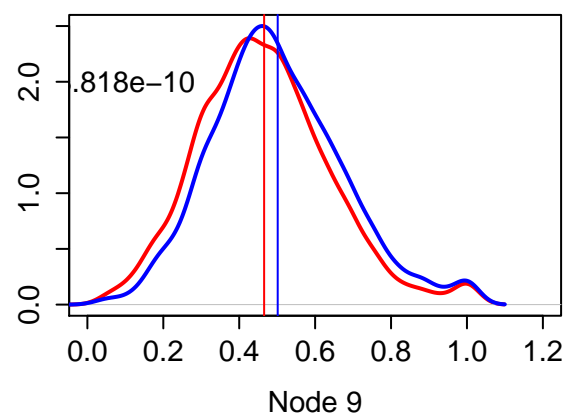

Supplement: Additional file 4 — Figure S3. Density plots of the proportion of nodes that have a bootstrap greater than 50 for homologs in the Hymenoptera dataset that are concordant with the node in question (blue) and those that are in conflict (red). Node numbers correspond to those in Fig. 2 in the main text. [file 12862_2015_423_MOESM4_ESM.pdf]

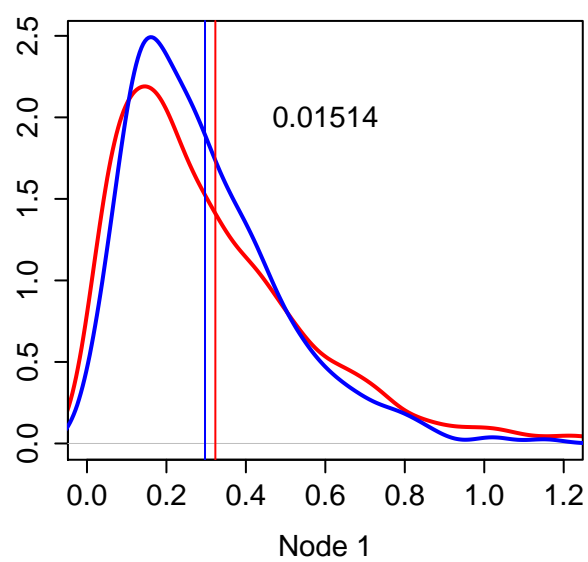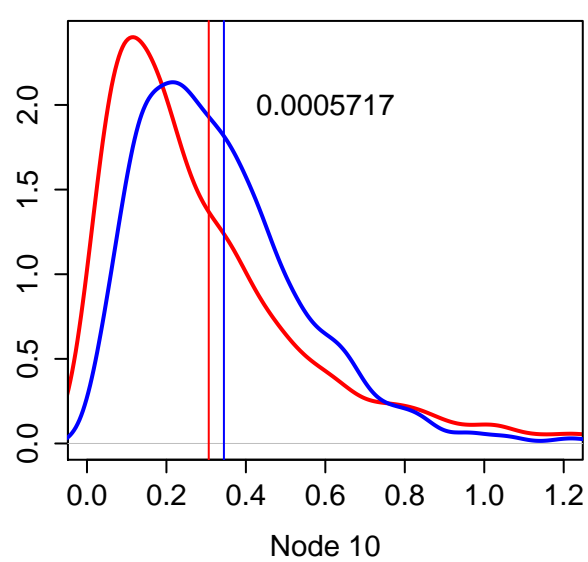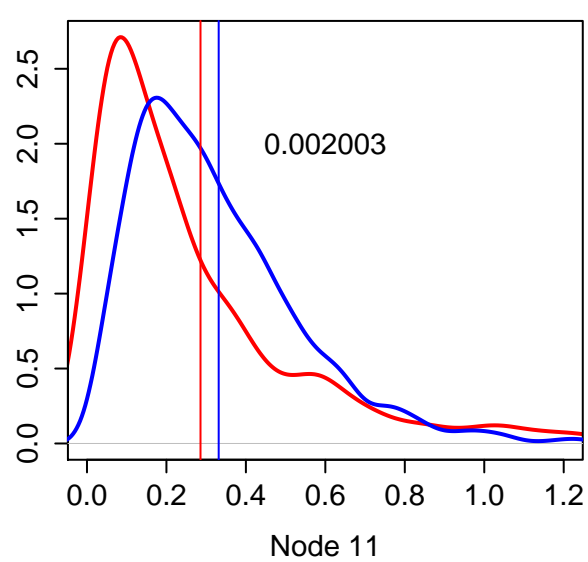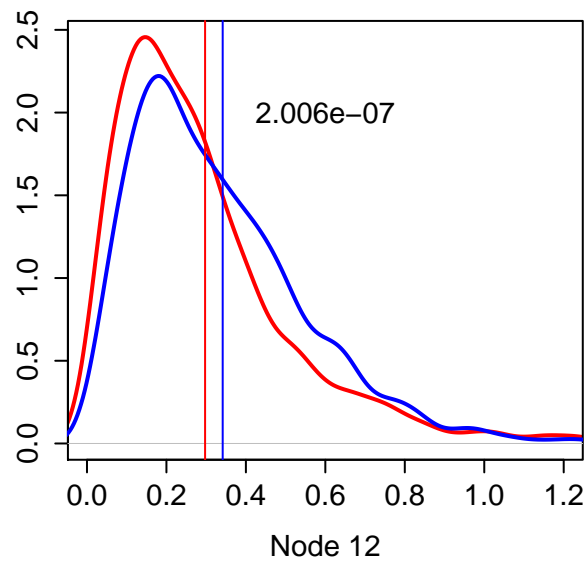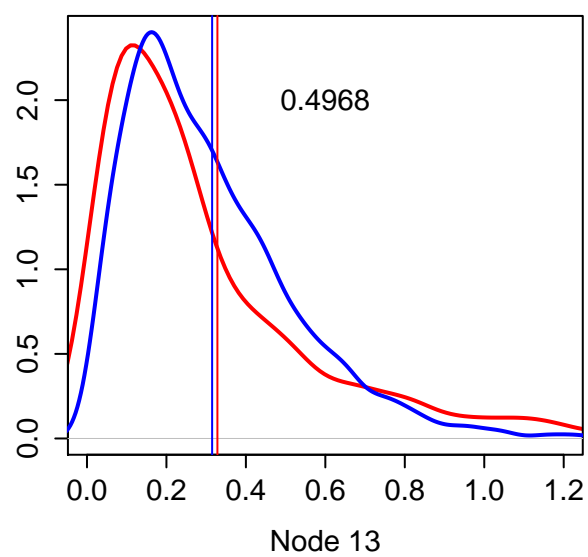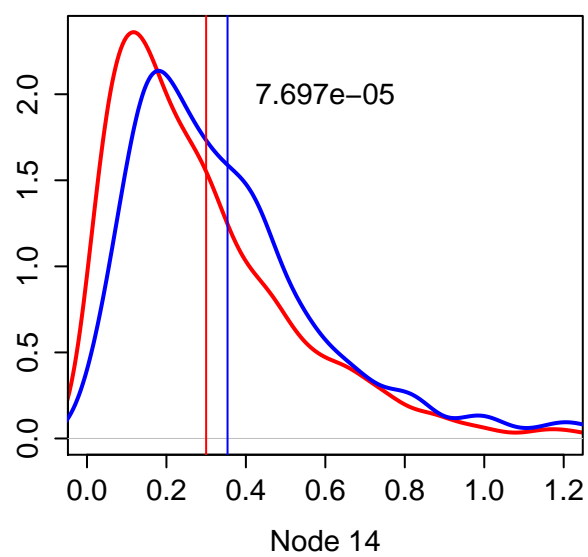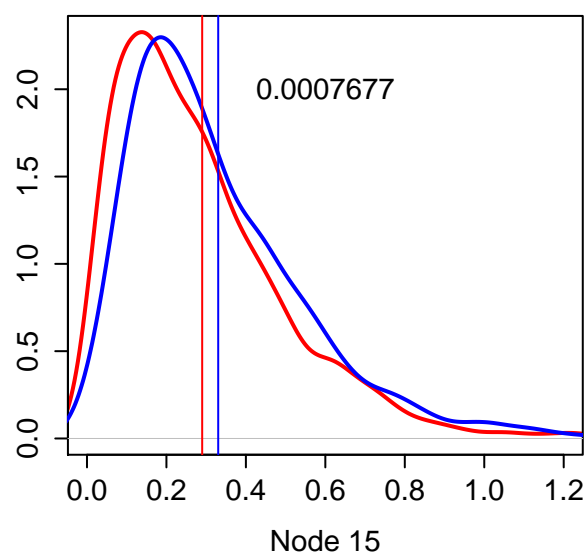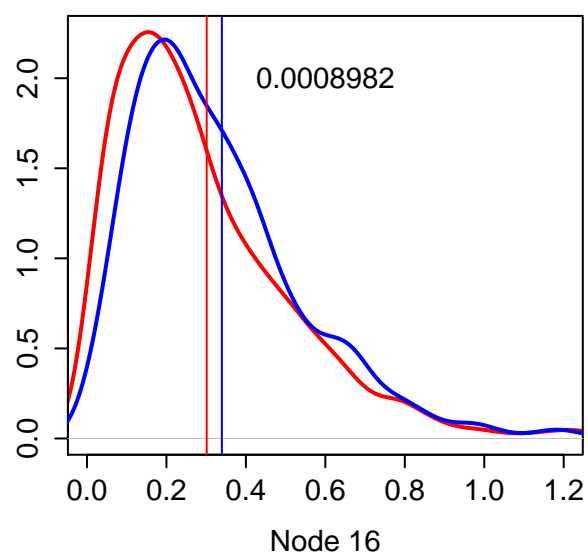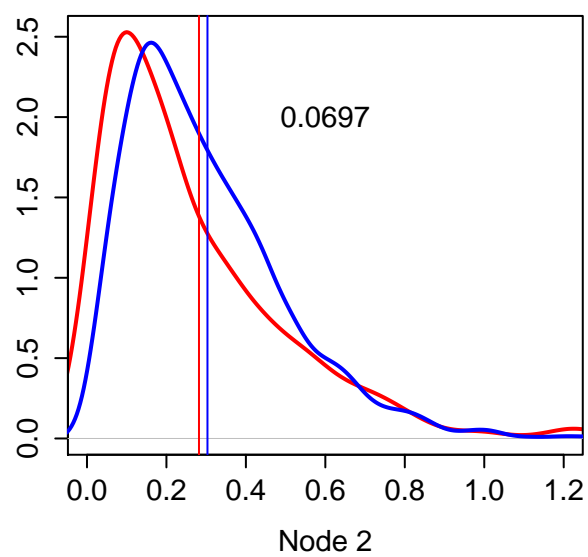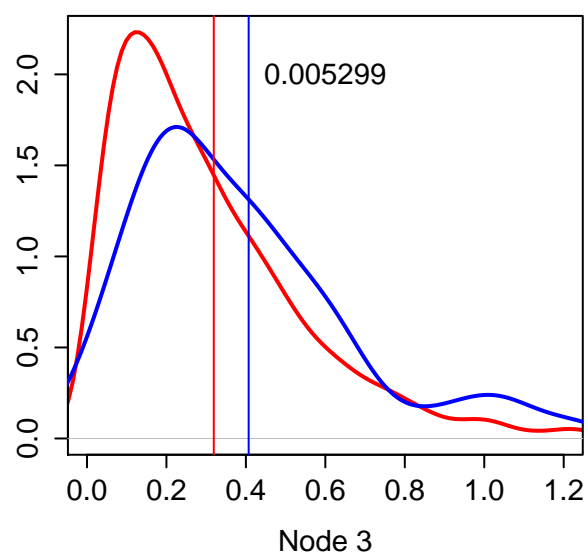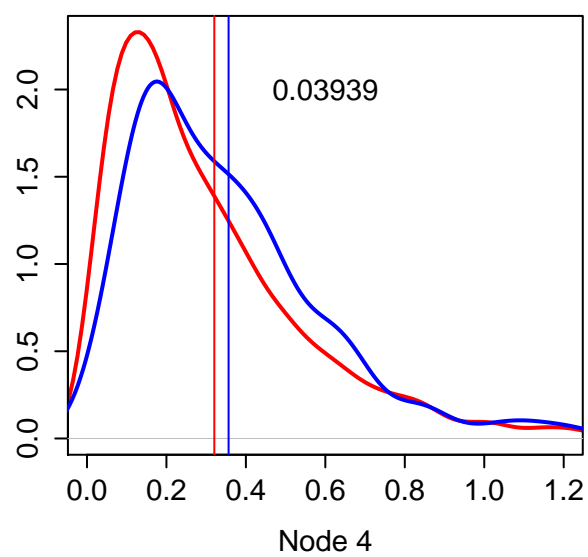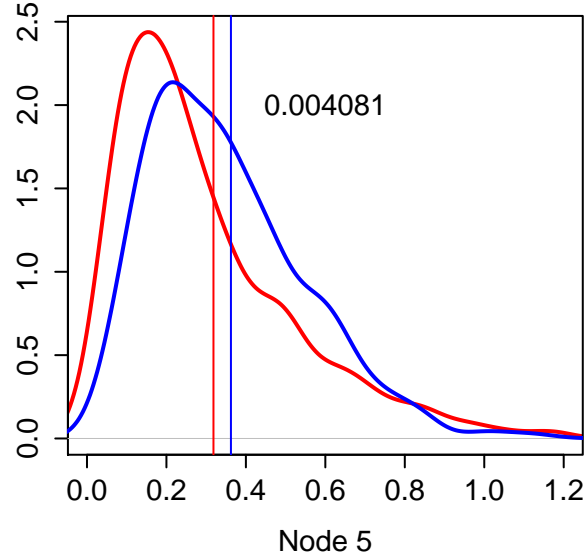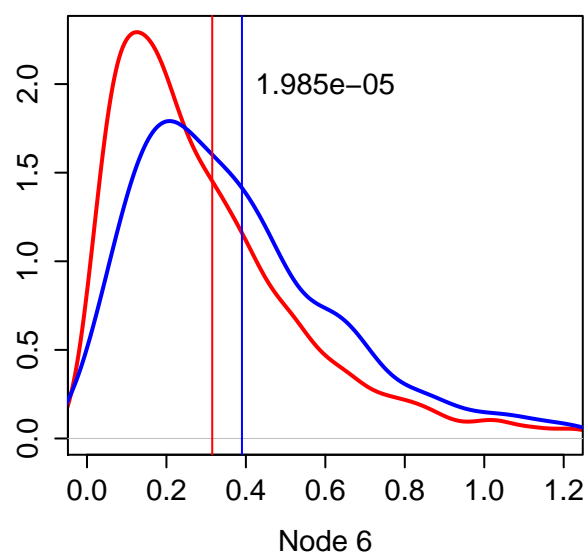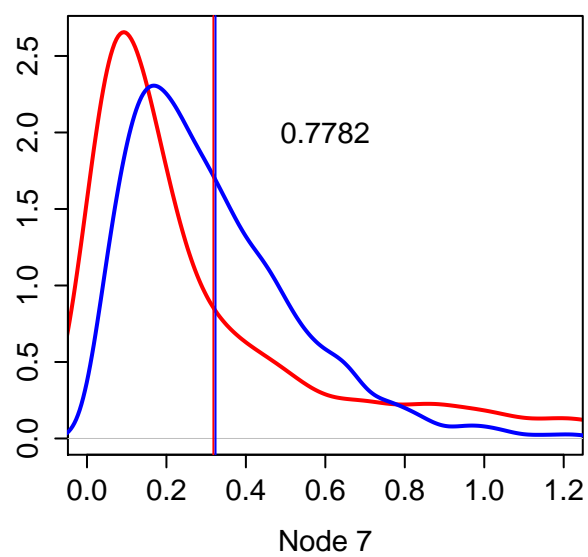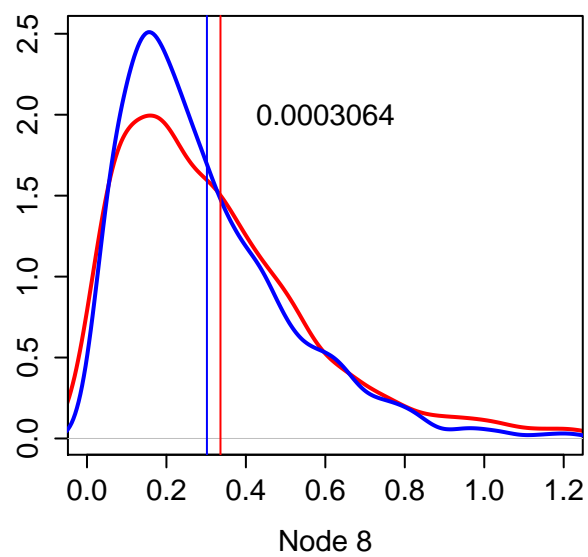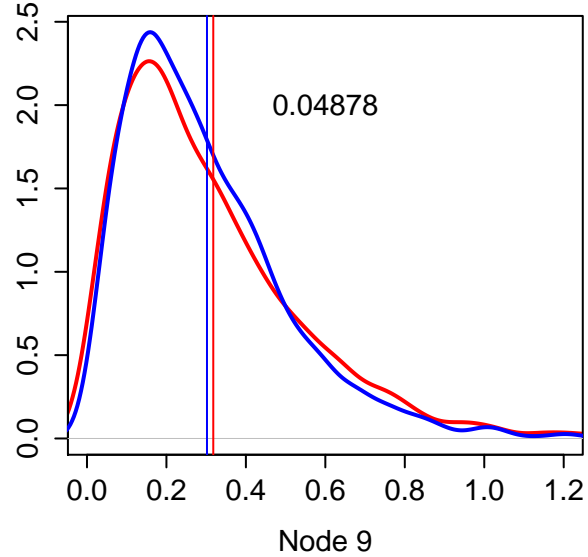

Supplement: Additional file 5 — Figure S4. Density plots of the average root to tip rates of molecular evolution for homologsin the Hymenoptera dataset that are concordant with the node in question (blue) and those that are in conflict (red). Node numbers correspond to those in Fig. 2 in the main text. [file 12862_2015_423_MOESM5_ESM.pdf]

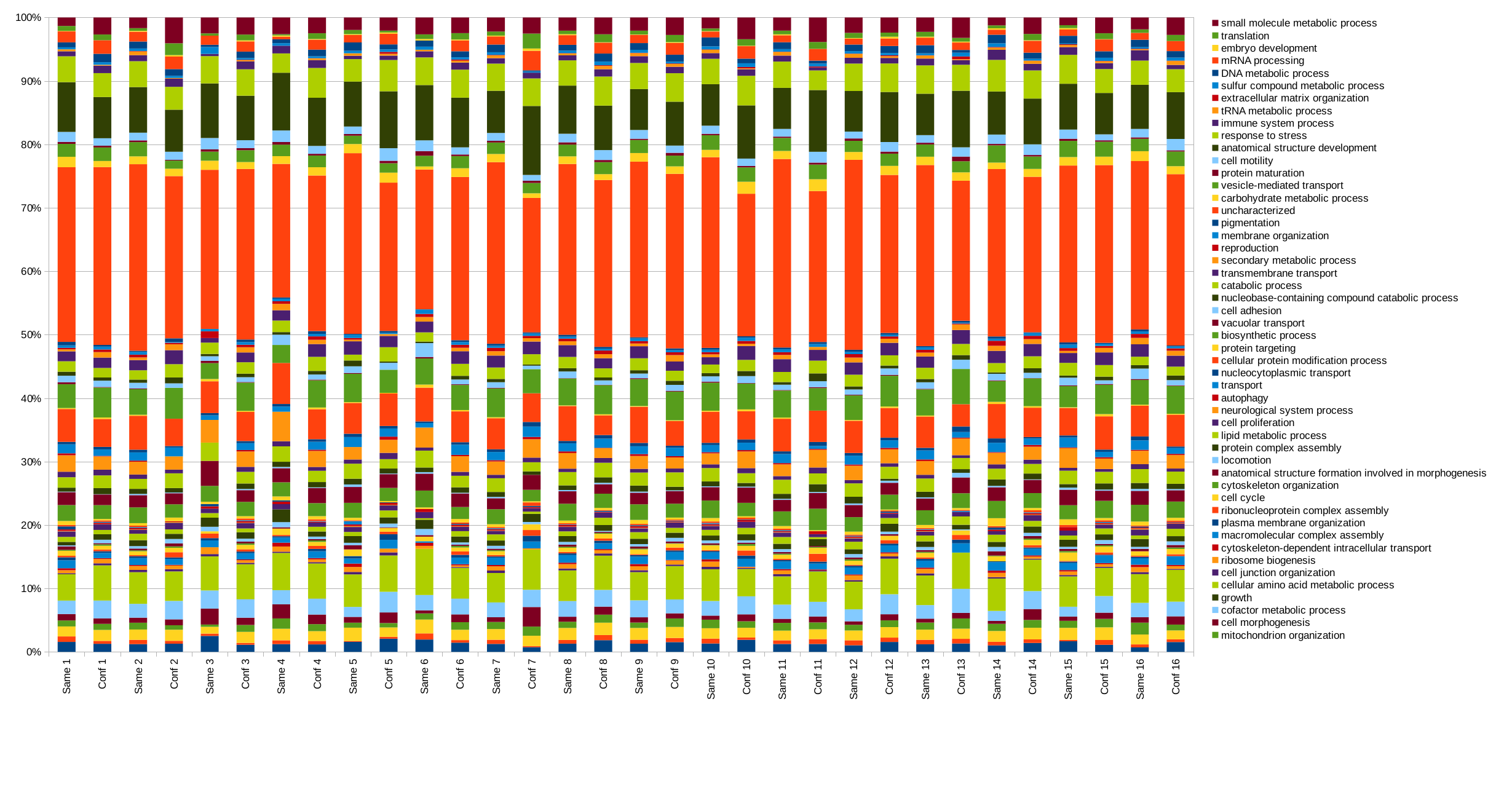

Supplement: Additional file 6 — Figure S6. The distribution of gene ontologies for homologs in the Hymenoptera dataset that are concordant with the node in question and those that are in conflict with the node in question. Node numbers correspond to those in Fig. 2 in the main text. [file 12862_2015_423_MOESM6_ESM.png]

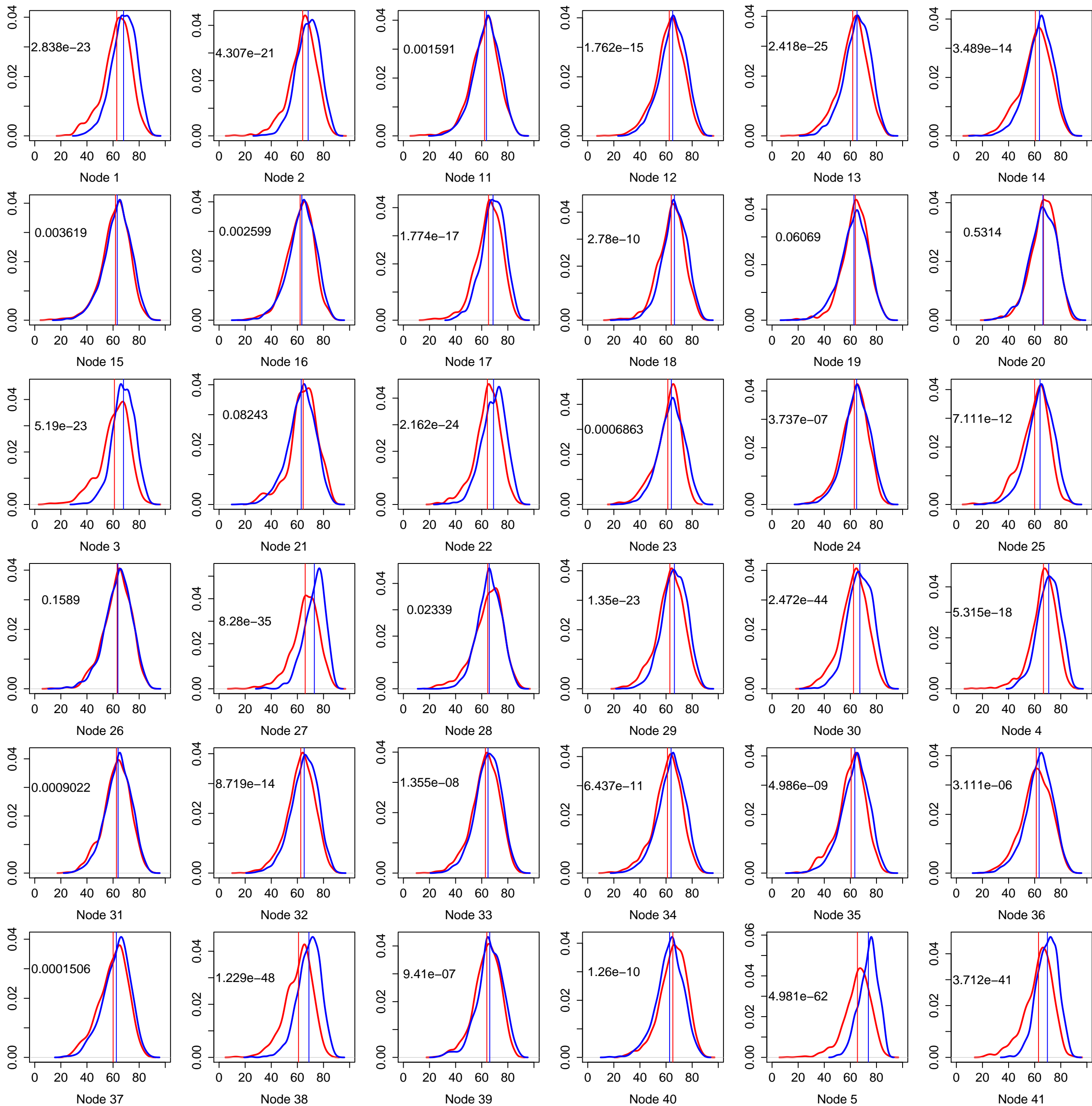

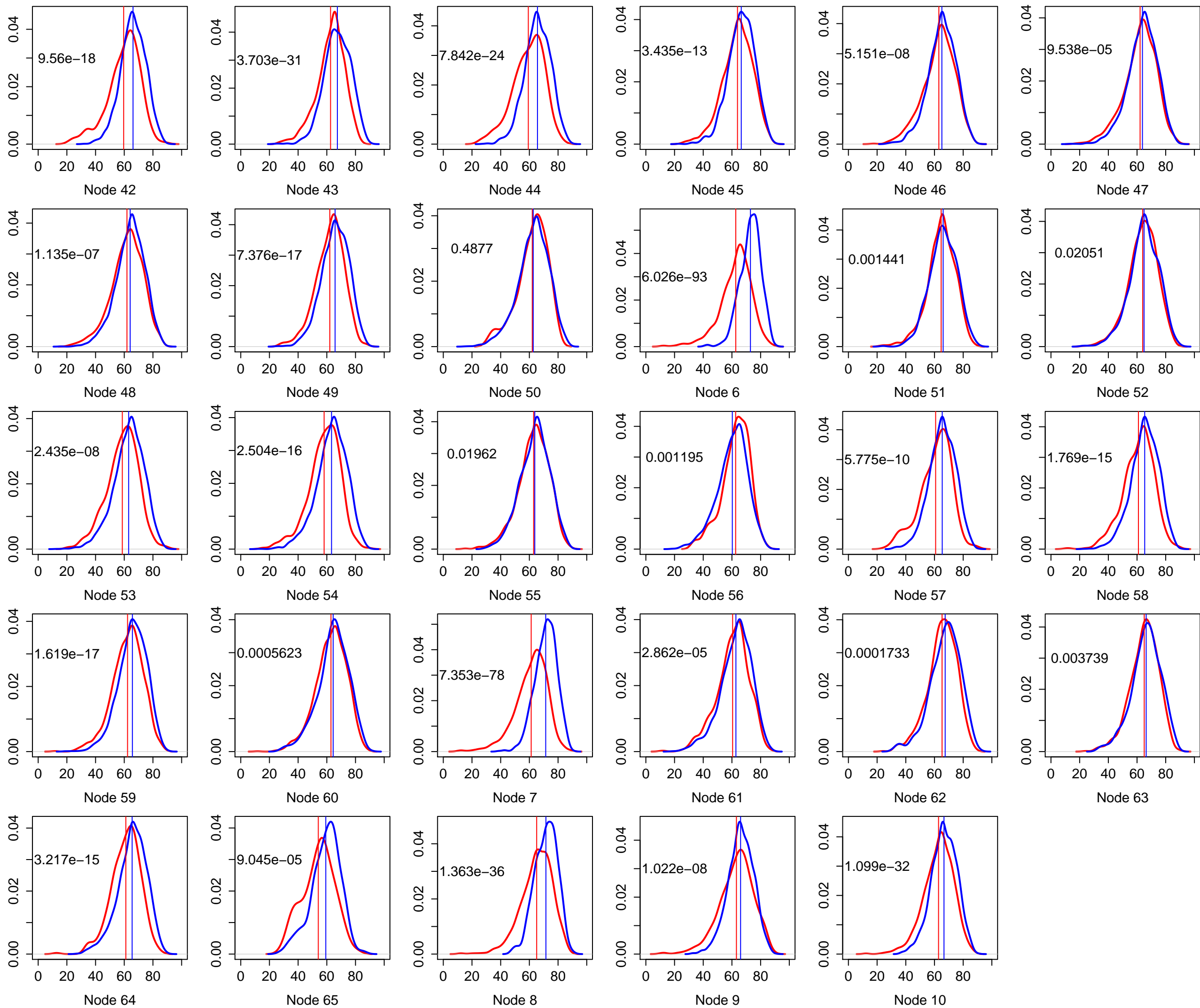

Supplement: Additional file 8 — Figure S7. Density plots of the average bootstrap values for homologs in the Caryophyllales dataset that are concordant with the node in question (blue) and those that are in conflict (red). Node numbers correspond to those in Fig. 4 in the main text. [file 12862_2015_423_MOESM8_ESM.pdf]

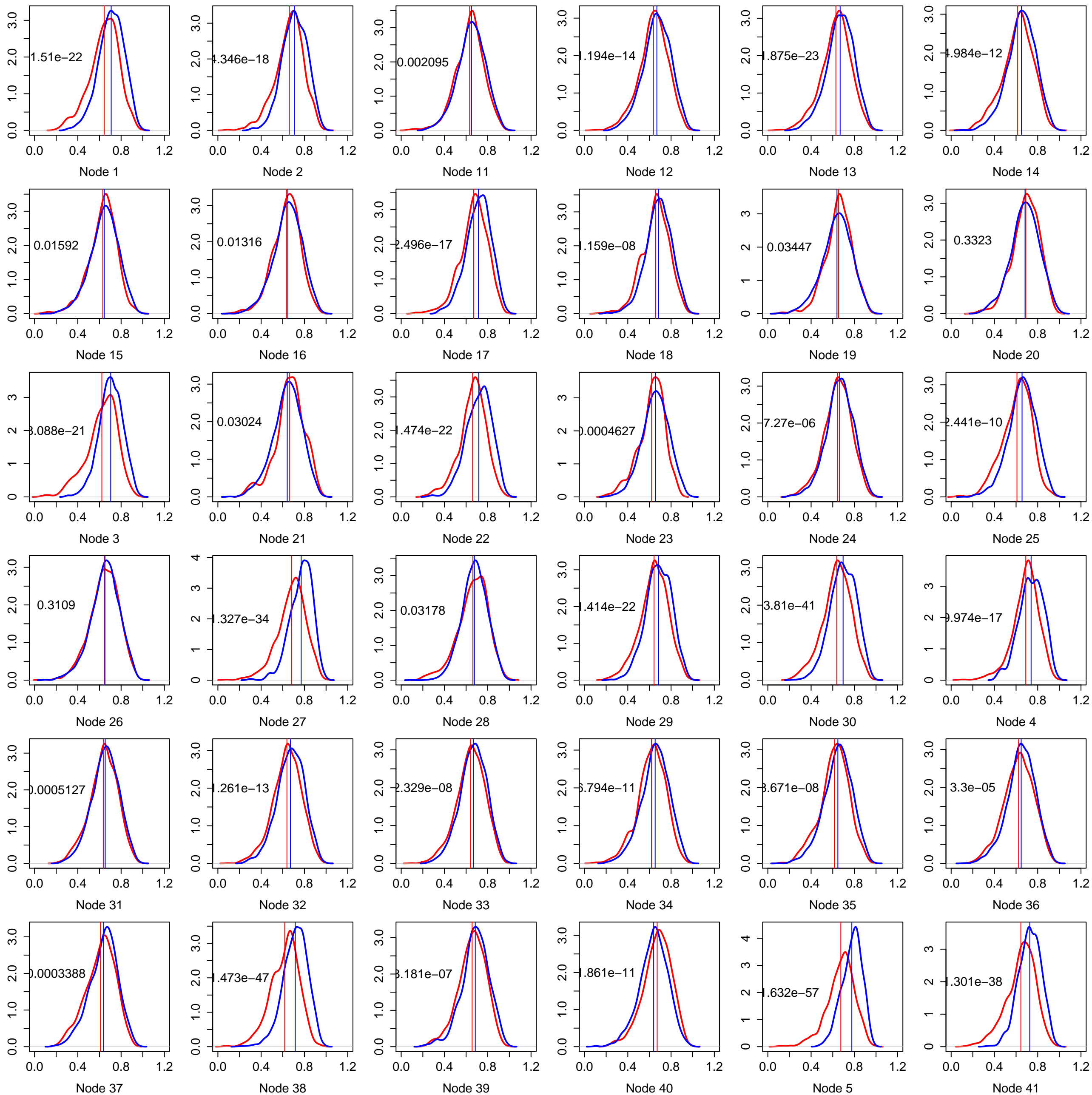

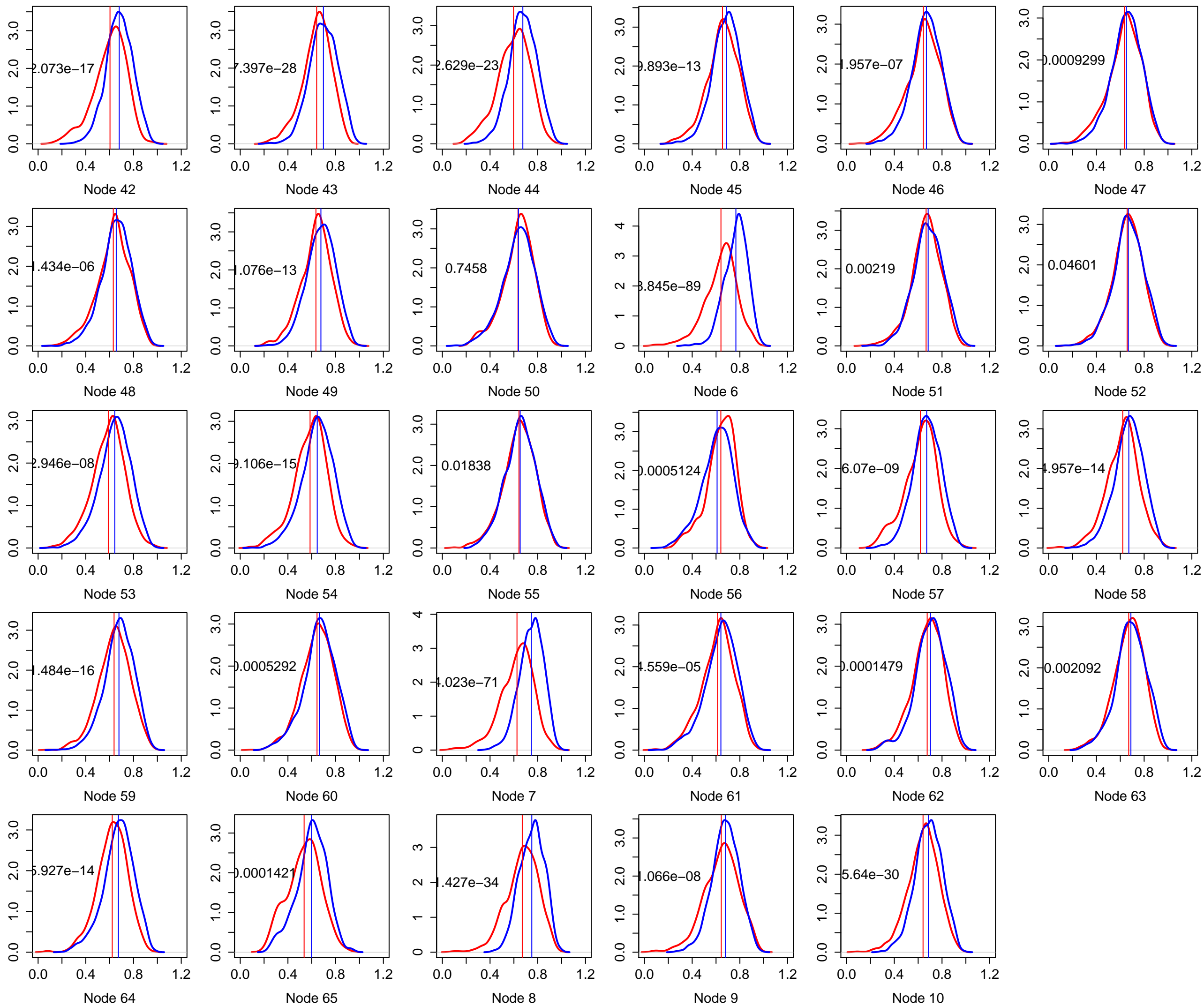

Supplement: Additional file 9 — Figure S8. Density plots of the proportion of nodes that have a bootstrap greater than 50 for homologs in the Caryophyllales dataset that are concordant with the node in question (blue) and those that are in conflict (red). Node numbers correspond to those in Fig. 4 in the main text. [file 12862_2015_423_MOESM9_ESM.pdf]

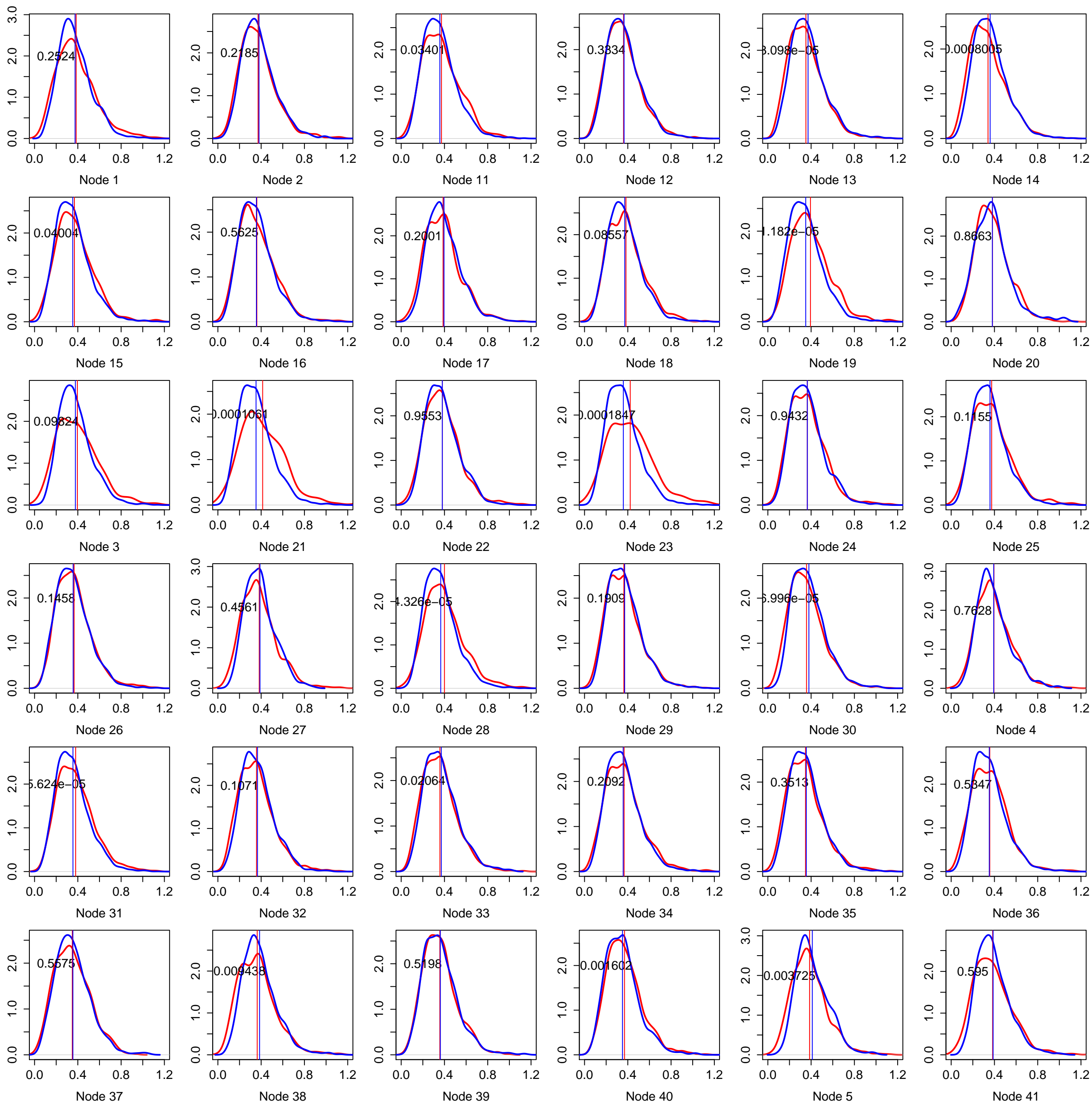

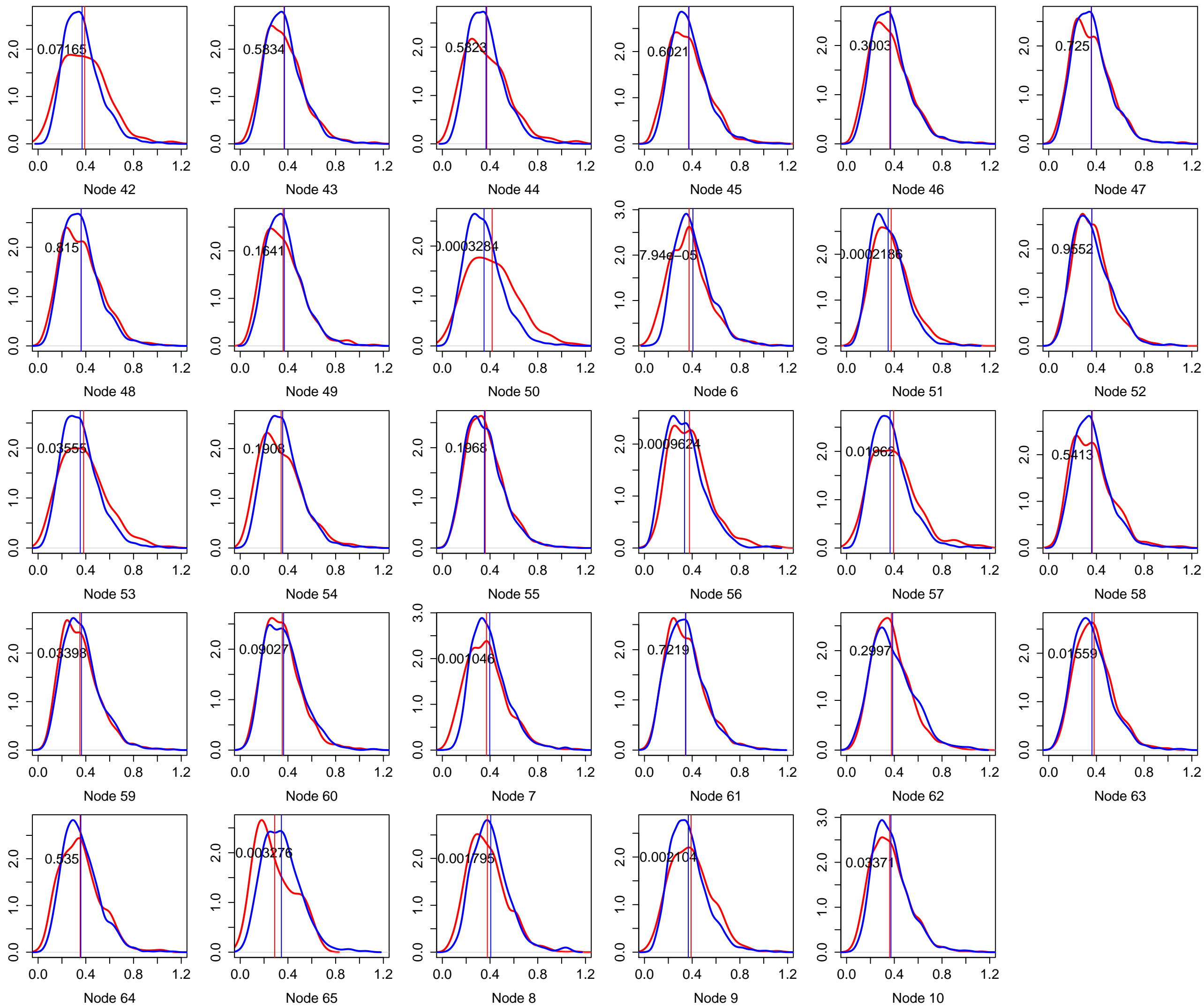

Supplement: Additional file 10 — Figure S9. Density plots of the average root to tip rates of molecular evolution for homologs in the Caryophyllales dataset that are concordant with the node in question (blue) and those that are in conflict (red). Node numbers correspond to those in Fig. 4 in the main text. [file 12862_2015_423_MOESM10_ESM.pdf]

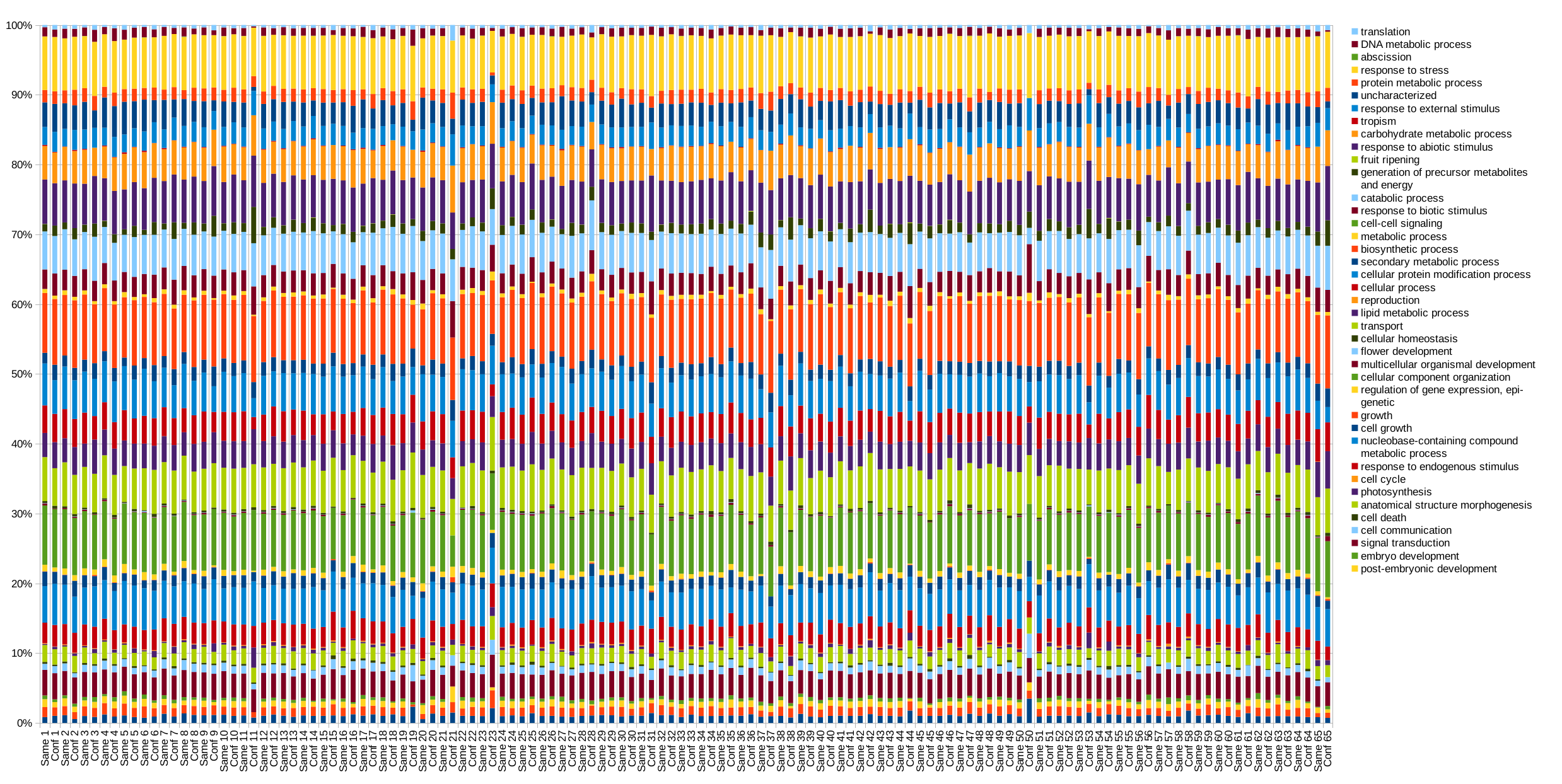

Supplement: Additional file 11 — Figure S11. The distribution of gene ontologies for homologs in the Caryophyllales dataset that are concordant with the node in question and those that are in conflict with the node in question. Node numbers correspond to those in Fig. 4 in the main text. [file 12862_2015_423_MOESM11_ESM.png]
