# Supplementary material for: Analysis of phylogenomic datasets reveals conflict, concordance, and gene duplications with examples from animals and plants
Source: BMC Evol Biol. 2015 Aug 5;15:150. doi: 10.1186/s12862-015-0423-0 (PMC4524127; doi:10.1186/s12862-015-0423-0)
Supplement: Additional file 7 — Figure S10. The proportion of the total homologs in the Caryophyllales dataset that support each conflicting alternative resolution, sorted from largest to smallest. Grey lines represent distributions based on coalescent simulations. Node numbers correspond to those in Fig. 4 in the main text. [file 12862_2015_423_MOESM7_ESM.pdf]

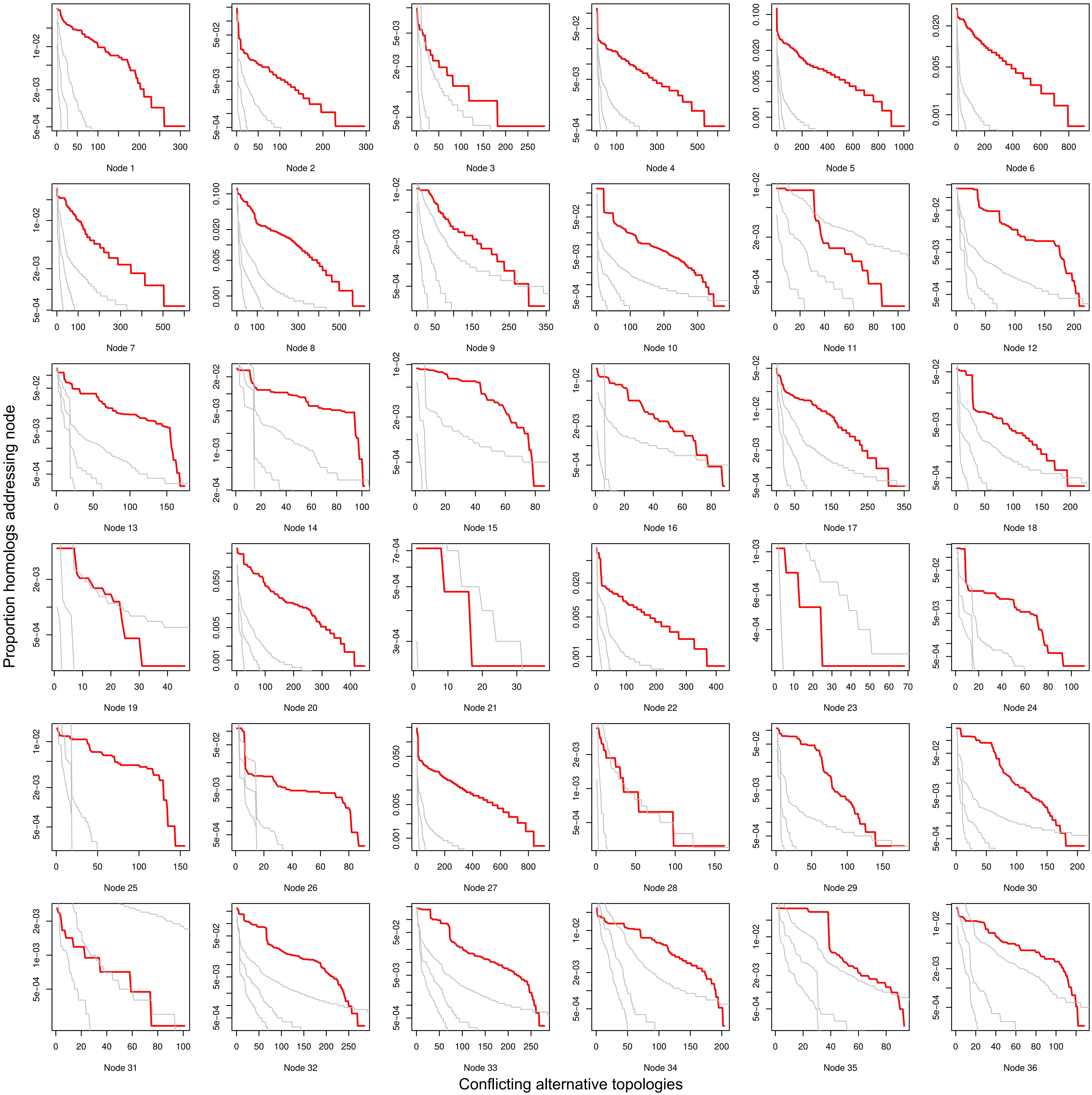
Proportion homologs addressing node
Conflicting alternative topologies
Node 1
Node 2
Node 3
Node 4
Node 5
Node 6
Node 7
Node 8
Node 9
Node 10
Node 11
Node 12
Node 13
Node 14
Node 15
Node 16
Node 17
Node 18
Node 19
Node 20
Node 21
Node 22
Node 23
Node 24
Node 25
Node 26
Node 27
Node 28
Node 29
Node 30
Node 31
Node 32
Node 33
Node 34
Node 35
Node 36

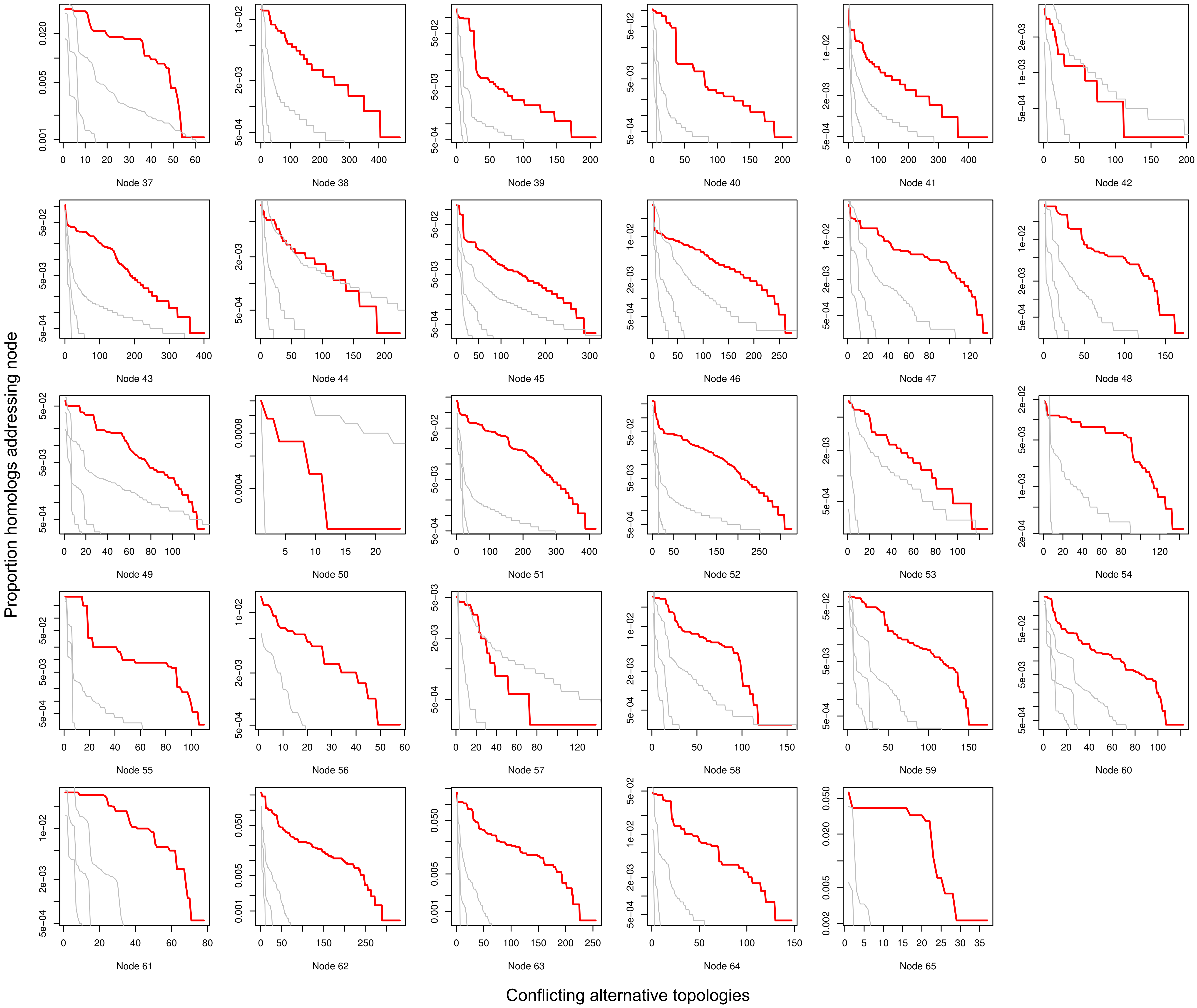

Proportion homologs addressing node
Conflicting alternative topologies
Node 37
Node 38
Node 39
Node 40
Node 41
Node 42
Node 43
Node 44
Node 45
Node 46
Node 47
Node 48
Node 49
Node 50
Node 51
Node 52
Node 53
Node 54
Node 55
Node 56
Node 57
Node 58
Node 59
Node 60
Node 61
Node 62
Node 63
Node 64
Node 65
